# Xylose utilization stimulates mitochondrial production of isobutanol and 2-methyl-1-butanol in *Saccharomyces cerevisiae*

**DOI:** 10.1186/s13068-019-1560-2

**Published:** 2019-09-20

**Authors:** Yanfei Zhang, Stephan Lane, Jhong-Min Chen, Sarah K. Hammer, Jake Luttinger, Lifeng Yang, Yong-Su Jin, José L. Avalos‬

**Affiliations:** 1 Department of Chemical and Biological Engineering, Princeton University, 101 Hoyt Laboratory, William Street, Princeton, NJ 08544 USA; 2 Andlinger Center for Energy and the Environment, Princeton, NJ USA; 3 Department of Molecular Biology, Princeton University, Princeton, NJ USA; 4 Carl R. Woese Institute for Genomic Biology, University of Illinois at Urbana-Champaign, Urbana, IL USA; 5 Department of Food Science and Human Nutrition, University of Illinois at Urbana-Champaign, Urbana, IL USA; 6 Lewis Sigler Institute for Integrative Genomics, Princeton University, Princeton, NJ USA; 7 Department of Chemistry, Princeton University, Princeton, NJ USA

**Keywords:** Isobutanol, Xylose, 2-Methyl-1-butanol, Branched-chain higher alcohols, *Saccharomyces cerevisiae*, Mitochondrial engineering

## Abstract

**Background:**

Branched-chain higher alcohols (BCHAs), including isobutanol and 2-methyl-1-butanol, are promising advanced biofuels, superior to ethanol due to their higher energy density and better compatibility with existing gasoline infrastructure. Compartmentalizing the isobutanol biosynthetic pathway in yeast mitochondria is an effective way to produce BCHAs from glucose. However, to improve the sustainability of biofuel production, there is great interest in developing strains and processes to utilize lignocellulosic biomass, including its hemicellulose component, which is mostly composed of the pentose xylose.

**Results:**

In this work, we rewired the xylose isomerase assimilation and mitochondrial isobutanol production pathways in the budding yeast *Saccharomyces cerevisiae*. We then increased the flux through these pathways by making gene deletions of *BAT1*, *ALD6*, and *PHO13*, to develop a strain (YZy197) that produces as much as 4 g/L of BCHAs (3.10 ± 0.18 g isobutanol/L and 0.91 ± 0.02 g 2-methyl-1-butanol/L) from xylose. This represents approximately a 28-fold improvement on the highest isobutanol titers obtained from xylose previously reported in yeast and the first report of 2-methyl-1-butanol produced from xylose. The yield of total BCHAs is 57.2 ± 5.2 mg/g xylose, corresponding to ~ 14% of the maximum theoretical yield. Respirometry experiments show that xylose increases mitochondrial activity by as much as 7.3-fold compared to glucose.

**Conclusions:**

The enhanced levels of mitochondrial BCHA production achieved, even without disrupting ethanol byproduct formation, arise mostly from xylose activation of mitochondrial activity and are correlated with slow rates of sugar consumption.

## Background

**Fig. 1:**
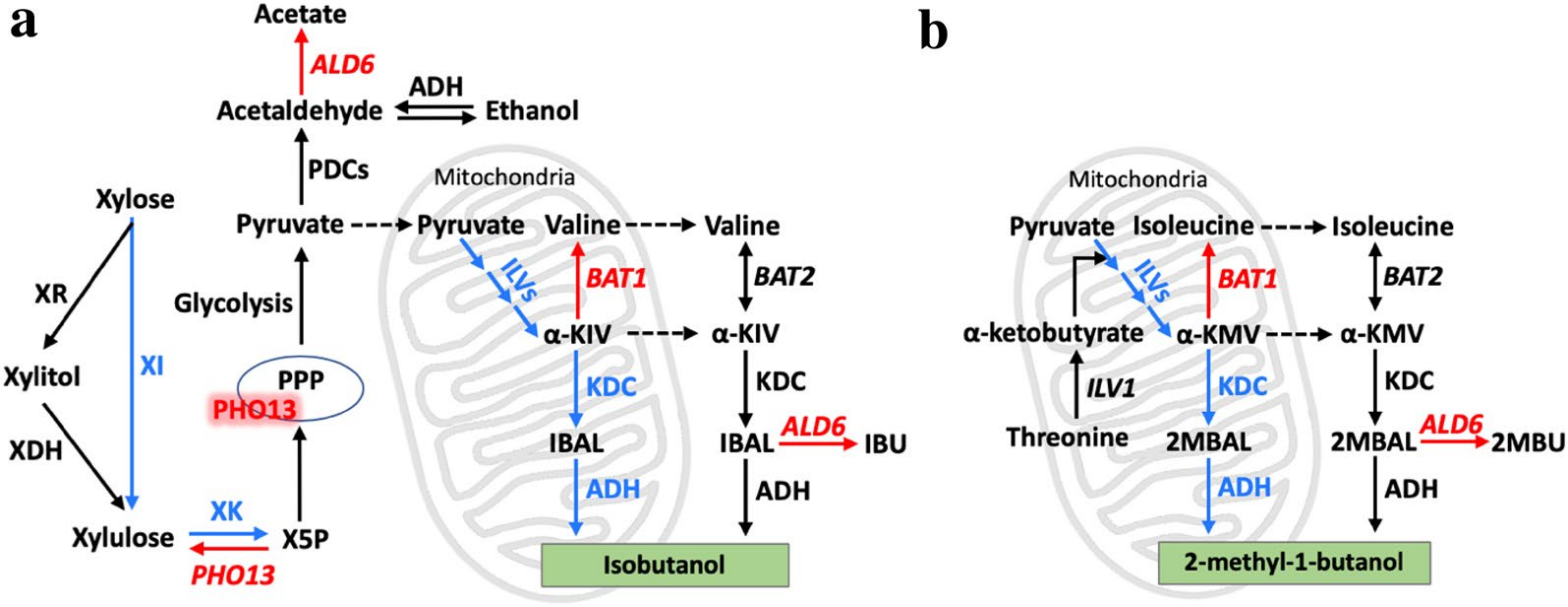
Engineering the mitochondrial isobutanol biosynthetic pathway in a xylose-utilizing strain of *S. cerevisiae*. Two different heterologous xylose utilization pathways have been used in yeast to convert xylose to xylulose: the isomerase pathway (used in this study), which uses xylose isomerase (XI); and the oxidoreductase pathway, consisting of xylose reductase (XR) and xylitol dehydrogenase (XHD). In both pathways, xylulose is subsequently phosphorylated to xylulose-5-phosphate (X5P) by xylulokinase (XK), and then channeled to glycolysis through the non-oxidative pentose phosphate pathway (PPP). **a** Mitochondrial isobutanol biosynthesis involves an upstream pathway that consists of the *ILV* genes including acetolactate synthase (*ILV2*), ketol-acid reductoisomerase (*ILV5*), and dihydroxyacid dehydratase (*ILV3*), as well as a downstream pathway that consists of mitochondrially targeted α-ketoacid decarboxylases (KDC) and alcohol dehydrogenases (ADH). **b** There is a considerable overlap between the upstream pathways for isobutanol and 2-methyl-1-butanol production, except the isoleucine precursor α-keto-β-methylvalerate (α-KMV) is synthesized by Ilv2p from one pyruvate and one α-ketobutyrate produced from threonine deamination catalyzed by threonine deaminase (*ILV1*); from there, the downstream Ehrlich degradation pathway for both branched-chain alcohols is identical. Genes overexpressed in our strains are shown in blue, while genes deleted are shown in red. *ALD6*: cytosolic aldehyde dehydrogenase, *BAT1*: mitochondrial branched-chain amino acid aminotransferase, *BAT2*: cytosolic branched-chain amino acid aminotransferase, PDCs: pyruvate decarboxylases, *PHO13*: alkaline phosphatase, α-KIV: α-ketoisovalerate, IBAL: isobutyraldehyde, IBU: Isobutyrate, α-KMV: α-keto-β-methylvalerate, 2MBAL: 2-methyl-1-butyraldehyde, 2MBU: 2-methyl-1-butyrate

Branched-chain higher alcohols (BCHAs), including isobutanol, isopentanol and 2-methyl-1-butanol (2-MbOH), are promising alternatives to the first-generation biofuel ethanol. These alcohols have better fuel properties than ethanol, such as higher energy density, ease of refining, and better compatibility with existing gasoline engines and infrastructures [1]. Several organisms have been engineered to produce isobutanol and other branched-chain alcohols by combining the biosynthetic and degradation pathways of branched-chain amino acids [2–10]. Isobutanol biosynthesis begins from pyruvate with acetolactate synthase (ALS), encoded in *Saccharomyces cerevisiae* by *ILV2*, followed by ketol-acid reductoisomerase (KARI), encoded by *ILV5*, and then dehydroxyacid dehydratase (DHAD), encoded by *ILV3* [11]. This upstream pathway results in the production of the valine precursor α-ketoisovalerate (α-KIV), which can be converted to isobutanol through the Ehrlich degradation pathway. This involves the decarboxylation of α-KIV by various α-ketoacid decarboxylases (α-KDCs), including those encoded by *PDC1* and *ARO10*, followed by reduction of the resulting isobutyraldehyde by various endogenous alcohol dehydrogenases (ADHs) (Fig. 1a). The 2-methyl-1-butanol biosynthetic pathway has considerable overlap with the upstream pathway of isobutanol biosynthesis and identical downstream Ehrlich degradation pathway. However, in this case the isoleucine precursor α-keto-β-methylvalerate (α-KMV) is synthesized by Ilv2p from one pyruvate and one α-ketobutyrate produced by threonine deaminase (*ILV1*) (Fig. 1b). In yeast, the gene products of the upstream pathway, *ILV2*, *ILV3*, and *ILV5* (collectively referred to as the *ILV* genes), are naturally located in mitochondria, where α-KIV is thus produced (*ILV1* and α-KMV are also mitochondrial). Yet the downstream pathway, composed of KDCs and ADHs, is naturally located in the cytosol. Alternative strategies have been used to overcome this physical fragmentation of the natural pathways, including colocalizing all enzymes in the cytosol [12], or in mitochondria [2].

To improve the environmental sustainability of biofuels, it is desirable to produce them from lignocellulosic biomass instead of starch or simple sugars [13–15]. Lignocellulosic biomass is primarily composed of three biopolymers: cellulose (~ 40–50%), hemicellulose (~ 25–35%) and lignin (~ 15–20%). The first two can be hydrolyzed into fermentable sugars: glucose from cellulose and mostly pentoses from hemicellulose [16, 17]. To enhance the economic viability of lignocellulosic biofuels, it is necessary to convert as much of the feedstock into valuable products, including the hemicellulose. As the primary component of hemicellulose, d-xylose (xylose) is the second most abundant sugar in lignocellulosic hydrolysates after glucose [18]. While yeast does not naturally assimilate xylose, several yeast strains have been developed to grow and produce ethanol from xylose [19–25].

Two different approaches have been taken to engineer xylose assimilation in yeast, both of which rely on converting xylose into xylulose-5-phosphate to feed glycolysis through the pentose phosphate pathway (PPP) (Fig. 1). In one strategy, called the isomerase pathway, xylose is isomerized to d-xylulose using a xylose isomerase (XI) [19, 21]. Alternatively, in the oxidoreductase pathway, xylose is converted to d-xylulose by sequential redox reactions carried out by xylose reductase (XR) and xylitol dehydrogenase (XDH), passing through xylitol as an intermediate [20, 22–25] (Fig. 1). In either strategy, the d-xylulose produced is phosphorylated to xylulose-5-phosphate by xylulokinase (XK), which feeds into the PPP.

Here, we describe a new strain engineered to produce isobutanol from xylose, which uses the mitochondrial isobutanol biosynthetic pathway [2] in a strain engineered with the isomerase xylose utilization pathway [26]. Previous efforts, using the isomerase xylose utilization and the cytosolic isobutanol biosynthetic pathways [27, 28], resulted in strains that produced up to approximately 110 mg/L of isobutanol [27]. Our strain produces as much as 3.10 ± 0.18 g/L of isobutanol as well as 0.91 ± 0.02 g/L of 2-MbOH, representing a 28-fold improvement from previously reported isobutanol titers, the highest xylose-derived isobutanol yields and productivities, as well the first report of 2-MbOH production from xylose. This study shows that xylose stimulates the activity of yeast mitochondria, thereby benefitting the mitochondrial isobutanol biosynthetic pathway for BCHA production from this pentose.

## Results

### Construction of a yeast strain to produce isobutanol from xylose

To develop *S. cerevisiae* strains that produce isobutanol from xylose, we introduced the mitochondrial isobutanol biosynthetic pathway [2] into the xylose-utilizing strain H145E10-XYLA3-1 (called Y58 in this study, Table 1) [19]. Y58 was evolved from a yeast strain engineered with the *Piromyces* xylose isomerase *XYLA*, and the xylulose kinase *XYL3* from *Pichia stipitis*, which together convert d-xylose to d-xylulose-5-P [19, 26]. The d-xylulose-5-P feeds into the glycolytic pathway through the non-oxidative pentose phosphate pathway (PPP), allowing the cell to metabolize xylose for cell growth and ethanol production [26] (Fig. 1). Using linearized plasmid pYZ34 (Table 2), we integrated the mitochondrial isobutanol pathway into genomic δ-sites (YARCdelta5) of Y58. This pathway consists of three genes for branched-chain amino acid biosynthesis (*ILV2*, *ILV5*, and *ILV3*, collectively referred to as the *ILV* genes), as well as genes encoding the Ehrlich degradation enzymes (CoxIV_MLS_-*ARO10* and CoxIV_MLS_-LlAdhA^RE1^) targeted to mitochondria using the *COXIV* mitochondrial localization signal (CoxIV_MLS_). This so-called mitochondrial isobutanol pathway boosts the production of branched-chain alcohols, relative to overexpressing the same enzymes in their native compartments [2]. After screening 20 colonies for isobutanol production in 48 h of high cell-density fermentation in 15% xylose we found that the best producer, YZy165 (Table 1), makes 162 mg/L of isobutanol, which is about sevenfold higher than the parental strain Y58 (24 ± 5 mg/L) (Additional file 1: Figure S1).

**Table 1:** Yeast strains used in this study

Strain	Description	Genotype	Source
Y58	Xylose-utilizing strain, H145E10-XYLA3-1, derived from H131-A3^CS^ (BF264-15Dau background), and then evolved	MATa, *leu2*-*3, 112*, URA3, *trp1*-*1∆::*(P_TDH3_-*RKI1*-T_CYC1_, P_TDH3_-*RPE1*-T_CYC1_, *TRP1*), *his2∆::*(P_TDH3_-*TKL1*-T_CYC1_, *HIS2*), *ade1∆::*(P_TDH3_-PsTAL1-T_CYC1_, *ADE1*), ChVI::(P_TDH3_-PsXYL3-T_CYC1_, 32 copies of P_TDH3_-PsXYLA-T_CYC1_), *arg4∆*::(*GRE3*^E193K^, *ARG4*)	[19, 26]
YZy165	Y58, δ-IbOH pathway	Y58 (δ-integration-*ILV2*, *ILV5*, *ILV3*, CoxIV_MLS_*ARO10, CoxIV*_*MLS*_LlAdhA^RE1^)	This study
YZy171	δ-IbOH pathway, *bat1Δ, ald6Δ, pho13Δ, ura3Δ*	YZy181 *ura3*-*K134stop*	This study
YZy173	δ-IbOH pathway, *bat1Δ*	YZy165 *bat1Δ*::hphMX	This study
YZy176	*bat1Δ, ald6Δ, pho13Δ, ura3Δ*	Y58 *bat1Δ*::hphMX, *ald6Δ*::kanMX, *pho13Δ*::natMX, *uar3*-*K134stop*.	This study
YZy177	δ-IbOH pathway, *bat1Δ, pho13Δ*	YZy165 *bat1Δ*::hphMX, *pho13Δ*::natMX	This study
YZy178	δ-IbOH pathway, *pho13Δ*	YZy165 *pho13Δ*::natMX	This study
YZy181	δ-IbOH pathway, *bat1Δ, ald6Δ, pho13Δ*	YZy165 *bat1Δ*::hphMX*, ald6Δ*::kanMX, *pho13Δ*::natMX	This study
YZy182	δ-IbOH pathway, *ald6Δ, pho13Δ*	YZy165 *ald6Δ*::kanMX, *pho13Δ*::natMX	This study
YZy183	δ-IbOH pathway, *ald6Δ*	YZy165 *ald6Δ*::kanMX	This study
YZy184	δ-IbOH pathway, *bat1Δ, ald6Δ*	YZy165 *bat1Δ*::hphMX, *ald6Δ*::kanMX	This study
YZy197	YZy171, pJA180	YZy171 (2μ-ILVs + CoxIV_MLS_LlKivd + CoxIV_MLS_LlAdhA^RE1^)	This study
YZy199	YZy171 (2µ_URA3_vector)	YZy171, pRS426	This study

**Table 2:** Plasmids used in this study

Plasmid	Description (brackets indicate reverse direction)	Source
pRS426	Amp^R^, 2μ, URA3	[65]
pJA180	(2μ-ILVs + CoxIV_MLS_-LlKivd + CoxIV_MLS_-LlAdhA^RE1^) Amp^R^, 2μ, URA3, P_TDH3_-*ScILV2*-HA-T_ADH1_-P_PGK1_-*ScILV3*-His-T_CYC1_-P_TEF1_-CoxIV_MLS_-LlAdhA^RE1^-cMycTag-T_ACT1_-[P_TDH3_-CoxIV_MLS_-LlKivd-cHATag- T_ADH1_-P_TEF1_-*ScILV5*-cMycTag-T_ACT1_]	[2]
pJA182	(2μ-ILVs + CoxIV_MLS_-*ARO10* + CoxIV_MLS_-LlAdhA^RE1^) Amp^R^, 2μ, URA3, P_TDH3_-*ScILV2*-HA-T_ADH1_-P_PGK1_-*ScILV3*-His-T_CYC1_-P_TEF1_-CoxIV_MLS_-LlAdhA^RE1^-cMycTag-T_ACT1_-[P_TDH3_-CoxIV_MLS_-*ScARO10*-cHATag-T_ADH1_-P_TEF1_-*ScILV5*-cMycTag-T_ACT1_]	[2]
pYZ17	Amp^R^, Lox71-kanMX-Lox66 gene-disruption cassette	This study
pYZ23	Amp^R^, Lox71-bleMX6-Lox66 δ-integration vector	[41]
pYZ34	(δ-integration-ILVs, CoxIV_MLS_-*ARO10*, CoxIV_MLS_-LlAdhA^RE1^) Amp^R^, δ-integration-Lox71-ShBle-Lox66-P_TDH3_-*ScILV2*-HA-T_ADH1_-P_PGK1_-*ScILV3*-His-T_CYC1_-P_TEF1_-CoxIV_MLS_-LlAdhA^RE1^-cMycTag-T_ACT1_-[P_TDH3_-CoxIV_MLS_-*ScARO10*-cHATag-T_ADH1_-P_TEF1_-*ScILV5*-cMycTag-T_ACT1_]	This study
pYZ55	Amp^R^, Lox71-hphMX-Lox66 gene-disruption cassette	This study
pYZ84	Amp^R^, Lox71-natMX-Lox66 gene-disruption cassette	This study

### Improvement of isobutanol production by targeted gene deletions

**Fig. 2:**
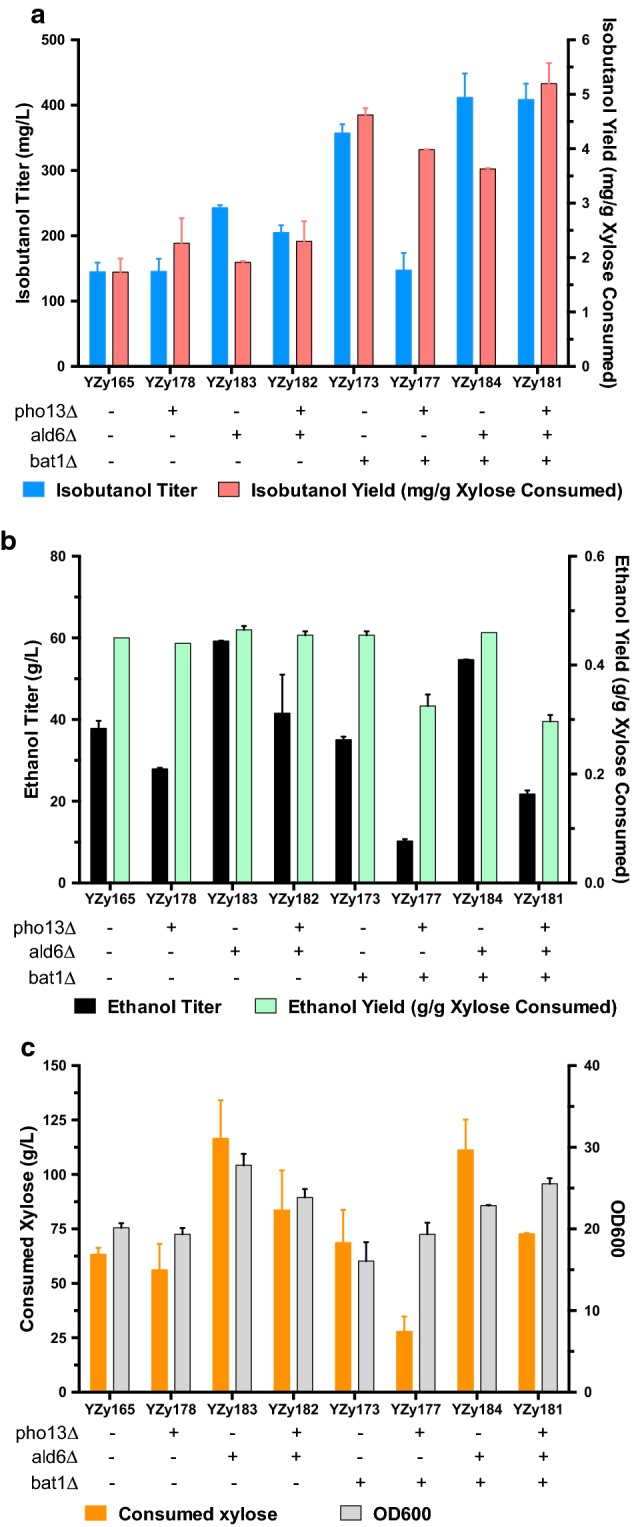
Effects of deleting *PHO13*, *ALD6* and *BAT1* in YZy165, on product formation and xylose consumption. **a** Effects on isobutanol titers and yields. **b** Effects on ethanol titers and yields. **c** Effects on xylose consumption and final OD_600_. Measurements taken from 72 h-long fermentations in 15% xylose. All data represent the mean ± SD of biological triplicates. Additional data monitored at different time points are shown in Additional file 1: Figure S2

Previous studies have shown that deletion of *PHO13* enhances xylose fermentation in *S. cerevisiae* [22, 29, 30]. *PHO13* encodes a promiscuous alkaline phosphatase that dephosphorylates metabolites and proteins. Although the precise role of this enzyme in boosting xylose utilization is not completely understood, it has been shown to have phosphatase activity on xylulose-5-phosphate [22] (Fig. 1). In addition, *PHO13* deletion causes transcriptional changes that affect the oxidative pentose phosphate pathway (PPP), other pathways that regenerate NADPH, and *TAL1*. The later encodes transaldolase, a bottleneck enzyme of the PPP whose activity, combined with that of Pho13p, leads to the accumulation of sedoheptulose as a byproduct [29]. When we deleted *PHO13* in YZy165 (YZy178, Table 1), we observed a modest increase in isobutanol yield (2.3 ± 0.5 mg/g xylose from 1.7 ± 0.3 mg/g xylose, Fig. 2a) and approximately 36% reduction in ethanol titers (27.9 ± 0.3 g/L from 37.8 ± 1.9 g/L, Fig. 2b) from 15% xylose in 72 h high cell-density fermentations. However, deletion of *PHO13* also results in a proportional reduction in xylose consumption (Fig. 2c), which leads to practically unchanged isobutanol titers (Fig. 2a and Additional file 1: Figure S2). The parental strain Y58 is already engineered to overexpress enzymes in the PPP (Table 1), which could explain the small effects on cell growth and isobutanol production of deleting *PHO13*.

The aldehyde dehydrogenase encoded by *ALD6* has been shown to divert the isobutyraldehyde precursor to isobutyrate at the expense of isobutanol production [31, 32] (Fig. 1). In addition, Ald6p is involved in converting the ethanol produced from fermentation to acetic acid, which reduces cell growth and product yields [33–35]. Disruption of *ALD6* in YZy165 (YZy183, Table 1) indeed leads to a 1.7-fold improvement in isobutanol production from xylose (Fig. 2a). Because Ald6p is a cytosolic enzyme, this result suggests that a fraction of isobutyraldehyde reduction may actually occur in the cytosol. This is consistent with the high activity of the mitochondrial α-KIV carrier, which transports α-KIV produced in mitochondria to the cytosol, where it can be decarboxylated by endogenous cytosolic ketoacid decarboxylases to isobutyraldehyde [36]. However, the largest effect of deleting *ALD6* is a substantial boost in cell growth measured by optical density (OD_600_, Additional file 1: Figure S2f), ethanol titers (Fig. 2b), and xylose consumption (Fig. 2c), suggesting that much of the improvement to isobutanol production may come from decreasing acetic acid production and boosting biomass formation. Moreover, deletion of both *ALD6* and *PHO13* (YZy182, Table 1) did not result in further increase in isobutanol production (Fig. 2a).

We also explored the effect of deleting the mitochondrial branched-chain amino acid transaminase encoded by *BAT1*, which converts the α-KIV precursor to valine exclusively in mitochondria (Fig. 1a). We found that deleting *BAT1* in YZy165 results in a strain (YZy173, Table 1) that produces 358 ± 13 mg/L isobutanol (from 15% xylose in 72 h high cell-density fermentations), which is a substantial improvement relative to deletions of *PHO13* (YZy178) or *ALD6* (YZy183) alone, and 2.5-times higher than the isobutanol produced by the parental YZy165 strain (Fig. 2a).

When we combined multiple deletions in the same strain (Table 1), we sometimes saw modest improvements in isobutanol production relative to the strain containing *BAT1* deleted (YZy173). Deleting both *BAT1* and *PHO13* (YZy177) produces the same titers as deleting *PHO13* alone (YZy178), which is less than half of what the *BAT1* deletion alone (YZy173) produces; yet the yield in the double knockout strain (YZy177) is 76% higher than that in the *ΔPHO13* strain (YZy178, Fig. 2a). The improvement in yield observed in YZy177 relative to YZy178 is a consequence of a substantial reduction in xylose consumption in YZy177 (Fig. 2c, Additional file 1: Figure S2), a trend perceived in the deletion of *PHO13* alone (when comparing YZy178 and the parental strain YZy165), which is exacerbated when combined with a *BAT1* deletion and ultimately explains the lower titers obtained with YZy177. When we deleted both *BAT1* and *ALD6* (YZy184), we saw an increase in isobutanol titers relative to the strain carrying only a *BAT1* deletion (YZy173) but also a lower yield (Fig. 2a, Additional file 1: Figure S2a, b), seemingly due to the accelerated xylose consumption observed in all strains with an *ALD6* deletion (Additional file 1: Figure S2e). However, the yields and titers of this double knockout are much higher than those of the strain with only *ALD6* deleted (YZy183). Finally, the strain carrying all three deletions (YZy181) achieves the same isobutanol titers (409 ± 25 mg/L) as the strain with only *BAT1* and *ALD6* (YZy184) and the highest overall yields after 72 h of fermentation (5.2 ± 0.4 mg/g), which are 2.8- and 3.0-fold higher than the parental strain (YZy165) (Fig. 2a, Additional file 1: Figure S2a, b). This suggests that the negative impact of *PHO13* deletion on xylose consumption is repealed by the additional deletion of *ALD6*, (consistent with the improvement in xylose consumption in YZy182 relative to YZy178), while preserving the yield enhancement brought also by the *PHO13* deletion. Nevertheless, the biggest strain improvement comes from deleting *BAT1*, which is a hallmark of mitochondrial isobutanol production [36].

### Improvement of isobutanol production with additional copies of the mitochondrial biosynthetic pathway

Considering the possibility that stronger overexpression of the isobutanol biosynthetic pathway could further improve production, we set out to introduce additional copies of the mitochondrial isobutanol pathway using 2μ plasmids. Because YZy181 is derived from Y58, a uracil prototrophic strain [19, 26], we first introduced a missense mutation in the *URA3* gene of YZy181 to make YZy171 (Table 1, see “Methods”) with a *ura3* mutant allele compatible with the *URA3* auxotrophic marker in our 2μ plasmids. Next, we transformed YZy171 with one of the three possible 2µ plasmids [2]: pJA182 (containing the *ILV* genes, CoxIV_MLS_-*ARO10*, and CoxIV_MLS_-LlAdhA^RE1^), pJA180 (containing the same genes, except CoxIV_MLS_-LlKivd, instead of CoxIV_MLS_-*ARO10*), or pRS426 (an empty plasmid control, Table 2). We also used these plasmids to transform YZy176, a strain derived from deleting *PHO13*, *ALD6*, and *BAT1* from Y58 and introducing a missense mutant *ura3* allele (as a control strain lacking the isobutanol biosynthetic pathway in δ-integration sites).

For each test strain transformation, we screened 22 colonies (3 for each empty plasmid control) for isobutanol production in 48 h high cell-density fermentations in 15% xylose. We found that most colonies of YZy171 transformed with 2μ plasmids containing biosynthetic pathways produce significantly higher isobutanol titers compared to colonies with empty plasmids (Additional file 1: Figure S3). This enhancement is more pronounced in some colonies harboring pJA180, with the best strain (isolated as YZy197, Table 1) producing over 1 g/L. Interestingly, the pJA180 plasmid contains a different α-KDC (CoxIV_MLS_-LlKivd) from the one introduced into δ-sites of YZy171 (CoxIV_MLS_-*ARO10*); thus, YZy197 overexpresses both α-KDCs. We also found increased isobutanol production in colonies of YZy176 (lacking α-KDC in its δ-sites) transformed with pJA180 or pJA182. However, strains transformed with either plasmid produce approximately equal levels of isobutanol, and about the same as strains of YZy171 transformed with pJA182 (containing the same α-KDC, CoxIV_MLS_-*ARO10*, introduced in its δ-sites). Therefore, our results suggest that having the two different α-KDCs overexpressed simultaneously in the same strain, YZy197, is beneficial for isobutanol production.

### Production of isobutanol and 2-methyl-1-butanol from different sugars in buffered media

**Fig. 3:**
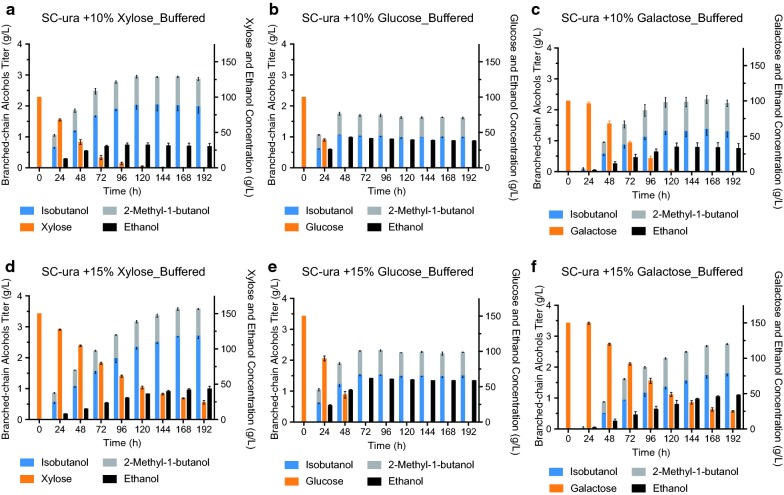
Time course of isobutanol and 2-methyl-1-butanol production of YZy197 in CaCO_3_-buffered media using different carbon sources. All data represent the mean ± SD of biological triplicates

We have previously shown that the mitochondrial isobutanol pathway can also lead to the conversion of glucose to other branched-chain higher alcohols (BCHAs) besides isobutanol, including 2-MbOH [2], which is another advanced biofuel (our strains are unable to produce isopentanol because of the *leu2*–*3* auxotrophic marker in their parent strain, Y58, Table 1). Therefore, we explored the ability of YZy197 to produce both isobutanol and 2-MbOH from xylose, as well as from glucose or galactose in CaCO_3_-buffered media (see “Methods”). Using buffered medium maintains the pH of fermentations slightly above pH 6, which substantially improves BCHA production (Additional file 1: Figure S4). We found that YZy197 can produce both isobutanol and 2-MbOH from all three sugars in buffered media (Fig. 3).

Our results show that YZy197 is more proficient at making BCHAs from xylose than from glucose or galactose. Fermentations in 10% xylose produce 2.05 ± 0.21 g/L of isobutanol and 0.91 ± 0.02 g/L of 2-MbOH (Fig. 3a), whereas in 10% glucose they produce 1.07 ± 0.01 g/L of isobutanol and 0.68 ± 0.05 g/L of 2-MbOH (Fig. 3b); and in 10% galactose they produce 1.32 ± 0.12 g/L of isobutanol and 0.93 ± 0.16 g/L of 2-MbOH (Fig. 3c). In addition, the rate of conversion of glucose is higher than that of xylose: 100% of the glucose load is consumed in approximately 48 h, while approximately 45% of xylose remains after the same period of time, and more than 120 h are needed to consume all the xylose. However, the titers of isobutanol and 2-MbOH obtained from 10% xylose in the first 48 h of fermentation are higher than those from glucose, or galactose in the same amount of time (Fig. 3a–c). This is consistent with the higher yields we obtain from xylose compared to other sugars (Fig. 3 and Additional file 1: Table S1).

### BCHA production in batch fermentations in different media

We carried out fermentations with YZy197 in SC-Ura medium containing different initial concentrations of xylose (4%, 8%, and 15%), glucose (15%) or galactose (15%) (Additional file 1: Figure S5 and Fig. 3). As expected, we found that higher concentrations of any sugar result in substantially higher isobutanol titers (Fig. 3 and Additional file 1: Figure S5), achieving the highest titer of 2.72 ± 0.10 g/L in 15% xylose (Fig. 3d). However, the titers of 2-MbOH are largely unchanged with increasing sugar concentrations (Additional file 1: Table S1), achieving similar values at 10% (0.91 ± 0.02 g/L) or 15% (0.86 ± 0.02 g/L) xylose. In addition, initial xylose consumption rates drop with increasing xylose concentrations and remain consistently low in fermentations starting at 15% xylose (Additional file 1: Figure S6a), which prevents full conversion even after 192 h (Fig. 3d). In contrast, increasing initial glucose concentrations raises its consumption rate (Additional file 1: Figure S7a).

**Fig. 4:**
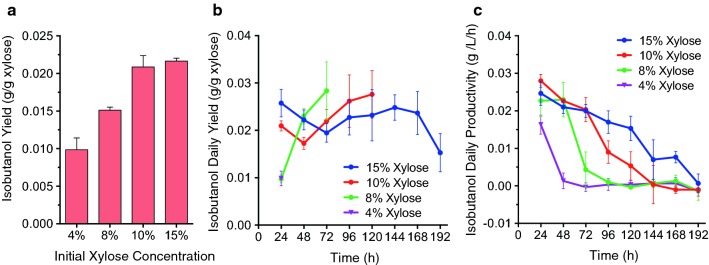
Effects of initial xylose concentration on isobutanol production in YZy197 fermentations. **a** Isobutanol overall yields at different xylose concentrations: *Y* = [IbOH]_final_/([Xyl]_initial_ − [Xyl]_final_). **b** Isobutanol daily yields at different xylose concentrations: *Y* = ([IbOH]_*t*=*i*_ − [IbOH]_*t*=*i*−1_)/([Xyl]_*t*=*i*−1_ − [Xyl]_*t*=*i*_). **c** Isobutanol daily productivities: *Y* = ([IbOH]_*t*=*i*_ − [IbOH]_*t*=i−1_)/24 h. [IbOH] = isobutanol concentration in mg; [Xyl] = xylose concentration in g; and *i* = time point in daily (24 h) allotments. All data represent the mean ± SD of biological triplicates

We also analyzed the isobutanol yields and productivities at different xylose concentrations. We found that higher xylose concentrations result in higher isobutanol yields (Fig. 4a), which correlate with decreased rates of xylose consumption (Additional file 1: Figure S6a, b), achieving a maximum overall yield of 23.0 ± 4.8 mg/g xylose in 15% xylose. Daily isobutanol yields start lower in fermentations starting with 8% or 10% xylose, but increase with time (Fig. 4b), (fermentations starting with 4% xylose ended before 24 h, so we could only measure one daily yield). On the other hand, fermentations starting with 15% xylose start with higher daily yields and remain relatively unchanged throughout most of the fermentation. Furthermore, initial isobutanol productivities are similar in all initial xylose concentrations in which they can be reliably measured (8%, 10%, and 15% xylose) but decline faster in fermentations at lower xylose concentrations (Fig. 4c); (again, we could not reliably measure productivities or consumption rates in fermentations starting with 4% xylose, because the substrate is fully consumed in less than 24 h). These results are thus consistent with our observation that reduced rates of xylose consumption at higher xylose concentrations result in increased isobutanol yields (Fig. 4a and Additional file 1: Figure S6).

We also tested YZy197 fermentations using Verduyn’s medium, a vitamin-enriched minimal medium that uses only ammonium salts as nitrogen source [37]. This medium has been effectively used for yeast xylose assimilation [38, 39] and isobutanol production from xylose [40]. Time course experiments (as described above), in 10 mL of CaCO_3_-buffered medium with 10% xylose (see “Methods”), revealed that YZy197 still produces significant amounts of isobutanol and 2-MbOH (Additional file 1: Figure S5c), although at slightly lower titers and productivities compared to those obtained in SC-Ura, 10% xylose (Fig. 3a). Therefore, YZy197 is effective at producing BCHAs from xylose in both synthetic-rich (SC-Ura) and minimal (Verduyn’s) media.

### Fed-batch fermentation for isobutanol production from xylose

To maximize isobutanol production from xylose, we carried out fed-batch fermentations at high sugar concentrations. Our results from batch fermentations revealed that higher xylose concentrations lead to higher isobutanol yields (Fig. 4a), productivities (Fig. 4c), and titers (Fig. 3d). Therefore, we set out to conduct fed-batch fermentations in which every 24 h we manually fed enough xylose to keep concentrations at approximately 10% or 15%, based on the calculated or measured xylose consumption rates (see “Methods”). The amount of xylose added in the first feeding at 24 h was calculated based on the xylose consumption rates obtained from batch fermentations (Additional file 1: Figure S6). The rest of the daily xylose feedings were calculated based on the measured rates of xylose consumption during the previous 24 h in the actual running fermentations. With this approach, we kept xylose concentrations at approximately 10% (103 ± 8 g/L, Additional file 1: Figure S8a) and 15% (154 ± 11 g/L, Additional file 1: Figure S8b) in fed-batch fermentations for 192 h.

**Fig. 5:**
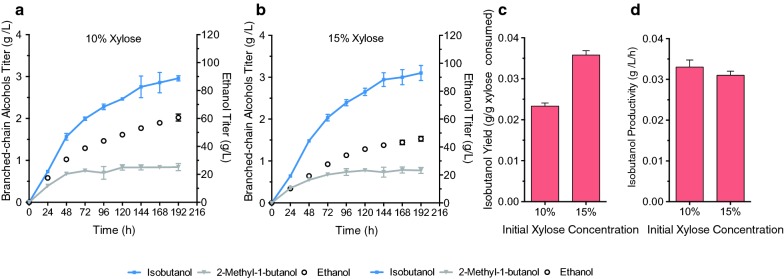
Fed-batch fermentation for isobutanol and 2-methyl-1-butanol production from xylose. Xylose was fed every 24 h to keep the concentration at 103 ± 8 g/L (**a**) or 154 ± 11 g/L (**b**). Maximum compounded isobutanol yields (**c**) and productivities (**d**) achieved after 48 h of fermentation. All data represent the mean ± SD of biological triplicates

Our results show that fed-batch fermentations in which xylose concentrations are kept high lead to improved isobutanol production (Fig. 5 and Additional file 1: Figure S8). YZy197 produces 2.96 ± 0.06 g/L of isobutanol in fermentations kept at approximately 10% xylose (Fig. 5a and Additional file 1: Figure S8c), and 3.10 ± 0.18 g/L of isobutanol (Fig. 5b and Additional file 1: Figure S8d) in those kept at 15% xylose, corresponding to as much as a 44% increase relative to batch fermentations (where xylose concentrations continuously drop). Even though the isobutanol titer is only improved by 5% in the higher xylose concentration, xylose consumption is lower at 15% xylose, which improves the yield. The maximum daily yield of 38.8 ± 2.4 mg/g xylose, achieved in the second day of fed-batch fermentations in 15% xylose, corresponds to 9.4% of the maximum theoretical yield (411 mg/g) and a 50% increase from the maximum daily yield achieved in batch fermentations starting with same amount of xylose (Table 3, Additional file 1: Table S1 and Figure S8). The compounded isobutanol yield of the fed-batch fermentation in 15% xylose during the first 96 h is thus higher than in any other fermentation (Additional file 1: Figure S8g), peaking after 48 h, when it reaches 35.8 ± 1.1 mg/g xylose, which is 74% higher than in the batch fermentation starting with the same xylose concentration (Fig. 5c).

**Table 3:** Highest titers, yields and productivities achieved with YZy197

YZy197	Titer^a^ (g/L)	Yield^a^ (mg/g xylose)	Productivity^b^ (mg/L/h)
Isobutanol	3.10 ± 0.18	38.8 ± 2.4	32.6 ± 1.7
2-Methyl-1-butanol	0.79 ± 0.07 (0.91 ± 0.02)^c^	18.4 ± 2.8	16.3 ± 1.4
Total branched-chain alcohols	3.89 ± 0.25	57.2 ± 5.2	48.9 ± 3.1

Values were obtained in CaCO_3_-buffered fed-batch fermentations in synthetic complete (minus uracil) medium, supplemented with ^a^150 g/L xylose, or ^b^100 g/L xylose

^c^The highest titer of 2-methyl-1-butanol was achieved in CaCO_3_-buffered batch fermentations in synthetic complete (minus uracil) medium, supplemented with 100 g/L xylose. Also see Additional file 1: Table S1

Isobutanol productivities also benefit from keeping xylose concentrations high in fed-batch fermentations (Additional file 1: Figure S8f, h, and Table S1), achieving the highest values again on the second day. The maximum daily productivities are similar for both 10% and 15% xylose fed-batch fermentations (34.6 ± 3.1 mg/L/h and 35.7 ± 2.4 mg/L/h, respectively, Additional file 1: Figure S8f), but the maximum compounded productivity is slightly higher in fed-batch fermentations in 10% xylose (32.6 ± 1.7 mg/L/h, Fig. 5d and Additional file 1: Table S1). The fact that the maximum values are achieved on the second day of fermentation is likely due to the adaptation period at the beginning of fermentation that takes place during the first day. Our results from fed-batch experiments are consistent with our findings in batch experiments that higher xylose concentrations boost isobutanol yields and productivities (Fig. 4a).

### Activation of mitochondrial activity by xylose

**Fig. 6:**
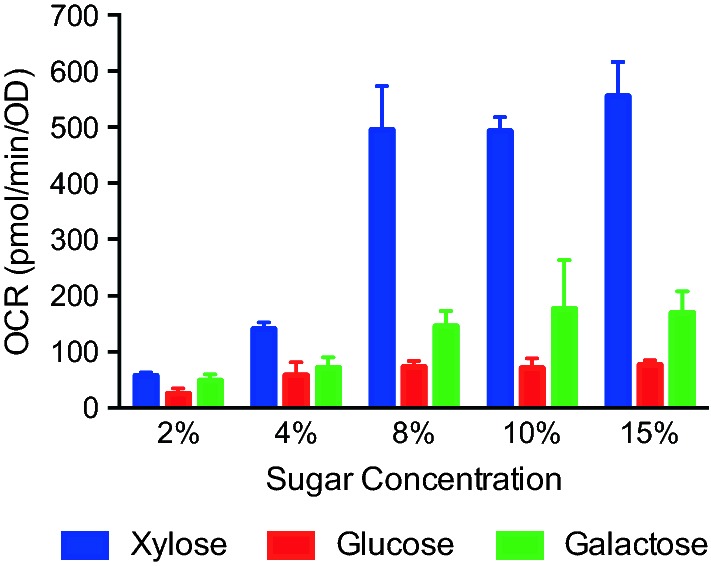
Oxygen consumption rate (OCR) of YZy197 in media supplemented with different carbon sources. Cells were harvested from cultures grown to mid-log phase in SC-Ura medium supplemented with different concentrations (2%, 4%, 8%, 10%, or 15%) of xylose, glucose, or galactose, respectively. The ORC values are calculated per OD_600_. All data represent the mean ± SD of biological triplicates

To test whether mitochondria are more active in xylose than in glucose or galactose, we carried out respirometry experiments to measure the oxygen consumption rate (OCR) of YZy197 in different concentrations of these sugars, ranging from 2 to 15%. We found that mitochondrial activity is indeed significantly higher in xylose than in glucose or galactose and that it is further stimulated with increasing sugar concentration (Fig. 6). At low sugar concentrations, the OCR is considerably lower, but even at 2% sugar, the OCR in xylose is twofold higher than in glucose. As sugar concentrations increase, so do the OCRs, but the rise in OCR is much more pronounced with increasing concentrations of xylose than of glucose or galactose. In fact, the OCR is 9.7-fold higher in 15% than in 2% xylose, while only 3- and 3.5-fold higher with the same increases in glucose or galactose, respectively (Fig. 6). Thus, the OCR in high (15%) sugar concentrations is 7.3 times higher in xylose than in glucose, and 3.3 times higher than in galactose. These results show that mitochondrial activity is significantly higher in xylose than in glucose or galactose, most notably at concentrations ranging from 8 to 15%, which could explain why mitochondrial isobutanol production is enhanced in xylose, especially at high concentrations.

## Discussion

We have engineered a xylose-utilizing yeast strain to produce BCHAs, including isobutanol and 2-MbOH, from this sugar. Yeast strains able to assimilate xylose have been previously engineered with the cytosolic isobutanol pathway [27, 28] to produce up to 110 mg/L of isobutanol from xylose [27]. In contrast, our strain engineered with the mitochondrial isobutanol pathway can produce as much as 3.10 ± 0.18 g/L of isobutanol and 0.91 ± 0.02 g/L of 2-MbOH. This is about a 28-fold improvement in isobutanol titers from the highest previous report, as well as the first account of a C5 BCHA (2-MbOH), produced from xylose (Table 3). Although our strain and those previously reported [27, 28] all utilize xylose via the xylose isomerase pathway, there are significant differences in their genetic backgrounds, as well as in the xylose isomerases and xylulokinases they use, which may account for some of the difference in isobutanol production between these strains. However, we speculate that mitochondrial compartmentalization of the isobutanol pathway is a key factor in the performance of our strain, especially in light of the stimulatory effect of xylose on mitochondrial activity. In a parallel study, we show that the mitochondrial isobutanol biosynthetic pathway introduced into another strain of equally divergent background, engineered to utilize xylose via the oxidoreductase pathway (instead of the isomerase pathway), can produce similarly high levels of isobutanol from xylose of up to 2.6 ± 0.1 g/L [40]. Thus, targeting the isobutanol biosynthetic pathway to mitochondria seems to be beneficial when producing BCHAs from xylose.

Mitochondrial production of BCHAs is higher in xylose than in other sugars. The parent strain Y58 was developed to efficiently grow and produce ethanol from xylose [19, 26], but it retains the ability to do the same in other sugars, including glucose and galactose. This gave us the opportunity to compare the performance of mitochondrial BCHA biosynthesis in these different sugars. Our results show that isobutanol and 2-MbOH titers and yields are, respectively, as much as 57% and 126% higher when produced from xylose (15%) than when produced from an equal amount of glucose; and as much as 33% and 30%, respectively, higher than when produced from the same amount of galactose (Additional file 1: Table S1). This suggests that mitochondrial metabolism is more active in xylose than in glucose, consistent with our observation that cells produce more ethanol from glucose than from xylose, which significantly impacts BCHA yields and titers [41].

There are different mechanisms by which xylose may enhance mitochondrial isobutanol production. We initially hypothesized that the reason could be related to the rate of sugar consumption, which is significantly lower in xylose compared to glucose (Fig. 3) and is inversely proportional to xylose initial concentrations (Fig. 3 and Additional file 1: Figure S6a, b) as well as isobutanol yields (Fig. 4). This hypothesis is consistent with observations we made in a separate study [40]. However, this trend does not occur in glucose or galactose (Additional file 1: Figure S6c, d), suggesting that an additional mechanism is involved during xylose assimilation. A more likely mechanism for the enhanced production of isobutanol in xylose is the higher mitochondrial activity observed in this sugar. Previous studies have shown that xylose is not recognized by *S. cerevisiae* as a fermentable carbon source the way glucose or galactose are [42–46]. Transcriptional data have also shown that xylose induces respiratory proteins [42]. Our own respirometry experiments confirmed that xylose enhances mitochondrial activity in YZy197 by more than sevenfold relative to glucose (Fig. 6). This stimulatory effect on mitochondrial activity is more pronounced at higher xylose concentrations, consistent with the enhanced isobutanol yields we observe in fed-batch fermentations in which we maintain high xylose concentrations (Fig. 5 and Additional file 1: Figure S8). Moreover, the reduced xylose consumption rate at higher xylose concentrations comes at the expense of reduced xylose fermentation, enabled by the higher amount of energy obtained from respiration, and consistent with the lower ethanol yields we observe. Therefore, it is likely that, by evading the Crabtree effect [29], xylose stimulates overall mitochondrial activity, including isobutanol production in this organelle. We anticipate that this effect of xylose on yeast metabolism will boost other metabolic pathways targeted to mitochondria [47].

The results obtained from the different gene deletion strains we tested reinforce the importance of mitochondrial activity in our strains. Deletion of *BAT1* produces by far the largest boost in production (Fig. 2). Not only does Bat1p withdraw α-KIV from isobutanol biosynthesis, but the valine produced by this competing reaction also interferes with the upstream BCHA biosynthetic pathway by inhibiting Ilv2p via Ilv6p [36]. Both of these inhibitory mechanisms of Bat1p occur exclusively in mitochondria; thus, the large increase in isobutanol production observed with its deletion alone strongly implicates mitochondrial activity in the biosynthesis of this alcohol in our strains. Once *BAT1* is deleted, the contributions from deleting *PHO13* and *ALD6* are marginal (only 14% higher than the *BAT1* deletion), again demonstrating the dominant role of mitochondrial activity in isobutanol production relative to the cytosolic Ehrlich pathway or even xylose assimilation efficiency.

We found that overexpressing two different α-KDCs (*ARO10* and LlKivd) in the same strain (YZy197) leads to about 30% higher isobutanol production than overexpressing either α-KDC alone at equal levels (Additional file 1: Figure S3). We hypothesize that heterodimer formation may lead to higher protein stability or enzymatic activity [48–50]. Another possibility is that having two genes encoding different α-KDCs increases the mRNA levels and thus enzyme concentration for this enzymatic step in the pathway [51–54]. These are intriguing possibilities, which we are currently exploring.

Our best strains developed in this study (YZy197), and a concurrent study (SR8-Iso) [40], achieve the highest reported levels of BCHA production from xylose. Nevertheless, their titers, yields, and productivities are insufficient for industrial application. The main reason is that most of the xylose assimilated by these strains is diverted to ethanol production. Thus, future work to remove or control ethanol biosynthesis will be key in developing industrially relevant strains to produce BCHAs from xylose. Deletion of the three pyruvate decarboxylase genes (*PDC1*, *PDC5*, and *PDC6*), which divert metabolic flux away from BCHAs and toward ethanol, results in strains that are notably difficult to work with because they lose the ability to grow on high glucose concentrations [55–57]. This complication may be avoided when cells grow on xylose instead of glucose, given that yeast does not recognize xylose as a fermentable carbon source [42]. If this is not the case, however, the timing and levels of *PDC1* expression may be controlled, for example optogenetically [41], in a xylose-utilizing strain, which would significantly improve BCHA production from xylose.

## Conclusions

In this study, we engineered a yeast strain that assimilates xylose via the xylose isomerase pathway with the mitochondrial isobutanol biosynthetic pathway. After deleting *PHO13*, *ALD6*, and *BAT1* to enhance xylose assimilation and isobutanol production, and introducing additional copies of the mitochondrial isobutanol pathway, we obtained a strain that produces as much as 3.10 ± 0.18 g/L of isobutanol and 0.91 ± 0.02 g/L of 2-MbOH from xylose (Table 3). This represents the highest isobutanol titer and yield ever reported from xylose (28- and 9.5-fold higher than previous reports [27], respectively.), as well as the first report of 2-MbOH production from this sugar. We provide evidence that mitochondrial activity is significantly higher in xylose than in glucose, probably because the Crabtree effect is averted due to the inability of yeast to recognize xylose as a fermentative carbon source, which likely stimulates mitochondrial isobutanol biosynthesis. Slow xylose consumption rates may also benefit mitochondrial isobutanol production. The effect of xylose on mitochondrial activity makes using the mitochondrial isobutanol biosynthetic pathway to produce BCHAs from xylose advantageous. Furthermore, the benefits that xylose brings to BCHA production in mitochondria will likely translate to other metabolic pathways targeted to this organelle.

## Methods

### Chemicals, reagents and general molecular biology methods

All chemicals and solvents were purchased from Sigma (St. Louis, Missouri, USA). Plasmid construction was performed through standard restriction enzyme cloning and isothermal assembly [58]. Target gene-disruption cassettes were amplified with polymerase chain reaction (PCR). Phusion High-Fidelity DNA Polymerase, Taq DNA polymerase, T4 DNA ligase, T5 Exonuclease, Taq DNA ligase, Calf Intestinal Alkaline Phosphatase (CIP), deoxynucleotide (dNTP) solution mix, and restriction enzymes were purchased from New England BioLabs (NEB, Ipswich, MA, USA) or Thermo Fisher Scientifics (Waltham, MA, USA). QIAprep Spin Miniprep, QIAquick PCR Purification, and QIAquick Gel Extraction Kits (Qiagen, Valencia, CA, USA) were used for plasmids isolation and DNA fragments purification according to the manufacturer’s protocols. The oligonucleotides used (Additional file 1: Table S2) were obtained from Integrated DNA Technologies (IDT, Coraville, Iowa, USA). The strain of *Escherichia coli* DH5α was used for routine construction and amplification of plasmids. All the constructed plasmids were verified by DNA sequencing (GENEWIZ, South Plainfield, NJ, USA).

### Plasmid construction

The plasmids used in this study are listed in Table 2. Three new PCR template plasmids containing gene disruption cassettes flanked by mutant loxP sites (lox71 and lox66) were constructed for gene deletion and drug-resistant marker recycling: pYZ17 (KanMX), pYZ55 (HphMX), pYZ84 (NatMX). Cre-mediated recombination between same oriented lox71 and lox66 of inserts derived from these plasmids results in deletion of the drug resistance marker and a defective double mutant loxP site (lox72) which has a very low affinity for Cre recombinase [59]. Plasmid pYZ17 was first constructed by replacing the two loxP sites in pUG6 [60] with mutant lox71 and lox66 sites using the isothermal assembly method [58]. Two overlapping DNA fragments were amplified from pUG6 using primer pairs Yfz_Oli67 & Yfz_Oli68, and Yfz_Oli69 & Yfz_Oli70, respectively (Additional file 1: Table S2). Plasmids pYZ55 and pYZ84 were constructed using restriction cloning by replacing the fragment between *Bgl*II and *Sac*I in pYZ17 with fragments cut with *Bgl*II and *Sac*I from pAG26 and pAG36 [61], respectively. The antibiotic resistant markers were amplified from these plasmids (pYZ17, pYZ55 and pYZ84) using a pair of primers containing the annealing sequences: primer 1 (5′-TACGCTGCAGGTCGACAACC-3′) and primer 2 (5′-CTAGTGGATCTGATATCACC-3′) with 5′ extensions containing 70 base pairs of homology to the sequences upstream and downstream of the ORF of the gene targeted for deletion.

We used a previously developed plasmid, pYZ23 [41], to target multiple copies of gene cassettes into genomic δ-sites YARCdelta5, the 337 bp long-terminal-repeat of *S. cerevisiae* Ty1 retrotransposons (YARCTy1-1, SGD ID: S000006792). The selection marker in pYZ23 is the shBleMX6 gene, which encodes a protein conferring resistance to zeocin and allows a selection of varying number of integration events based on varying zeocin concentrations. The level of zeocin resistance reflects the number of copies of the integration: resistance to higher concentration of zeocin correlates with a higher number of gene cassette copies integrated into δ-sites. The δ-integration plasmid pYZ34 (δ-integration of *ILV2*, *ILV5*, *ILV3*, CoxIV_MLS_-*ARO10*, and CoxIV_MLS_-LlAdhA^RE1^) was constructed by subcloning the gene cassette from previously described plasmid pJA182 [2] using restriction site pairs *Xma*I/*Asc*I (to extract gene cassettes) and *Mre*I/*Asc*I (to open pYZ23). Integration plasmid was linearized with *Pme*I prior to yeast transformation.

### Yeast strains, yeast transformation and growth media

All *S. cerevisiae* strains in this study (Table 1) were constructed from a xylose-utilizing strain Y58 (originally called H145E10-XYLA3-1), kindly provided by Dr. Gregory Stephanopoulos [19]. H145E10-XYLA3-1 (*MATa*, *leu2*-*3, 112*, *URA3*, *trp1*-*1∆::*(P_TDH3_-*RKI1*-T_CYC1_, P_TDH3_-*RPE1*-T_CYC1_, *TRP1*), *his2∆::*(P_TDH3_-*TKL1*-T_CYC1_, *HIS2*), *ade1∆::*(P_TDH3_-*PsTAL1*-T_CYC1_, *ADE1*), ChVI::(P_TDH3_-*PsXYL3*-T_CYC1_, 32 copies of P_TDH3_-*PsXYLA*-T_CYC1_), *arg4∆*::(*GRE3*^E193K^, *ARG4*)) is evolved from H131-A3^CS^, a strain previously engineered and evolved to assimilate xylose by overexpressing codon-optimized xylose isomerase (*XYLA*) from *Piromyces* sp., xylulokinase (*PsXYL3*) from *Pichia stipitis*, and the non-oxidative pentose phosphate pathway (PPP) [19, 26].

Deletions of *BAT1*, *ALD6,* and *PHO13* were obtained using PCR-based homologous recombination. DNA fragments containing lox71–lox66-flanked antibiotic resistance cassettes were amplified with PCR from pYZ55 (containing the hygromycin resistance gene hphMX4), pYZ17 (containing the G418 resistance gene KanMX), or pYZ84 (containing the nourseothricin resistance gene NAT1), using primers with 50–70 base pairs of homology to upstream and downstream of the ORF of the gene targeted for deletion. Transformation of gel-purified PCR fragments was done using the lithium acetate method [62]. Transformed cells were first plated onto nonselective plates with 10 g/L yeast extract, 20 g/L peptone, 0.15 g/L tryptophan and 20 g/L xylose (YPX) and grown overnight at 30 °C. Lawns were then replica-plated onto YPX plates with 300 µg/mL hygromycin (Invitrogen, Carlsbad, CA, USA), 200 µg/mL nourseothricin (WERNER BioAgents, Jena, Germany), or 200 µg/mL Geneticin (G-418 sulfate) (Gibco, Life Technologies, Grand Island, NY, USA), and grown for another 3 days at 30 °C until colonies appeared. All strains with gene deletions were genotyped with positive and negative controls to confirm the removal of the ORF of interest.

Integrations into genomic δ-sites were performed by transforming strains with *Pme*I-linearized pYZ34 and using the lithium acetate method [62]. Transformed cells were first incubated in YPX liquid medium for 6 h and then plated onto nonselective YPX agar plates for overnight growth. On the next day, the cells were replica-plated onto YPX agar plates with different concentrations (800, 1500 or 2000 µg/mL) of zeocin (Invitrogen, Carlsbad, CA, USA), and incubated at 30 °C until colonies appeared.

To restore the *ura3* auxotrophic marker in YZy181 and Y58 (to make YZy171 and YZy176), an 825-bp double-stranded DNA fragment of the ORF of *URA3* (orotidine-5′-phosphate decarboxylase) with three stop codons (taatgatag) inserted between Lys134 and Gln135 was synthesized from GENEWIZ (GENEWIZ, South Plainfield, NJ, USA) and transformed into the *URA3* allele. We then selected on 5-fluoroorotic acid (5-FOA, Zymo Research, Orange, CA, USA) for Ura^−^ strains.

Unless otherwise specified, yeast cells were grown on either YPX medium (10 g/L yeast extract, 20 g/L peptone, 0.15 g/L tryptophan and 20 g/L xylose) or synthetic complete (SC) drop-out medium (20 g/L glucose, 1.5 g/L yeast nitrogen base without amino acids or ammonium sulfate, 5 g/L ammonium sulfate, 36 mg/L inositol, and 2 g/L amino acid drop-out mixture).

### Yeast fermentations

High cell density fermentations were carried out in sterile 24-well microtiter plates (Cat. 229524, CELLTREAT Scientific Products, Pepperell, MA, USA) or in 50-mL conical tubes in an orbital shaker (Eppendorf, New Brunswick, USA) at 30 °C and at 200 rpm agitation. For plate fermentations, single colonies were first grown overnight in 1 mL of synthetic complete (SC) or synthetic complete minus uracil (SC-ura) medium supplemented with 2% xylose. The next day, 10 µL of the overnight culture was used to inoculate 1 mL of SC (or SC-ura) + 2% xylose medium in a fresh 24-well plate, and grown for 20 h. The following day, the plates were centrifuged at 1000 rpm for 5 min, the supernatant was discarded, and cells were re-suspended in 1 mL of SC (or SC-ura) supplemented with 15% xylose. The plates, in triplicates, were covered with sterile adhesive SealPlate^®^ sealing films (Cat. # STR-SEAL-PLT; Excel Scientific, Victorville, CA, USA) and incubated for 48 h, 72 h or 96 h, respectively, at 30 °C and with shaking at 200 rpm. The sealing film was used in all 24-well plate fermentations to keep semi-aerobic conditions in all wells and to prevent evaporation, “edge effects”, and cross-contamination between wells. At the end of the fermentations, the optical density at 600 nm (OD_600_) of the culture in each well was measured. Plates were then centrifuged for 5 min at 1000 rpm. The supernatant (approximately 1 mL) from each well was processed and analyzed using HPLC as described below.

Longer time course experiments (192 h) of high cell density fermentations were carried out semi-aerobically in sterile 50-mL conical tubes. Overnight cultures were prepared by inoculating 5 mL of liquid SC-ura medium supplemented with 2% xylose with a single colony from agar plates. The following day, 10 mL of liquid SC-ura medium supplemented with 2% xylose was inoculated with 100 µL of overnight cultures and grown for 20 h at 30 °C in 50 mL conical tubes. The next day, cell cultures were centrifuged for 5 min at 3000 rpm and re-suspended in 10 mL of SC-ura or Verduyn’s medium [37] minus uracil, supplemented with different amounts of xylose (4%, 8%, 10%, or 15%), glucose (10% or 15%) or galactose (10% or 15%). Dry autoclaved calcium carbonate (CaCO_3_) was added to 1% concentration to the resuspended culture to maintain a pH range of approximately 6.2–6.6 during the fermentation. We used CaCO_3_ as a pH buffering agent to prevent acidification during fermentation [63, 64]. Samples of 0.3 mL were taken at different time intervals during fermentation (0, 24, 48, 72, 96, 120, 144, 168, and 192 h), and processed for HPLC analysis as described below.

### Fed-batch fermentation

Fermentations were carried out in sterile 50-mL conical tubes, semi-anaerobically and with CaCO_3_ as described above. Starting 24 h after resuspending cells in fresh SC-ura media with 15% xylose, and every 24 h thereafter, xylose was added manually using concentrated xylose feed (50% xylose in SC-ura medium). The amount of xylose added the first 24 h was calculated based on the xylose consumption rate obtained from batch fermentation studies (Additional file 1: Figure S6). The amount of xylose added for the rest of the feedings was calculated based on the rate of xylose consumption during the previous 24 h for each individual fermentation, aiming to keep a xylose concentration of approximately 10% or 15% throughout the fermentation. Samples of 0.3 mL were taken at 0, 24, 48, 72, 96, 120, 144, 168, and 192 h for both OD_600_ measurements and HPLC analysis.

### Chemical analysis

The concentrations of xylose, ethanol, isobutanol, and 2-methyl-1-butanol were determined with high-performance liquid chromatography (HPLC) using an Agilent 1260 Infinity instrument (Agilent Technologies, Santa Clara, CA, USA). Samples were centrifuged at 13,300 rpm for 40 min at 4 °C to remove residual cells and other solid debris, and analyzed using an Aminex HPX-87H ion-exchange column (Bio-Rad, Hercules, CA, USA). The column was eluted with a mobile phase of 5 mM sulfuric acid at 55 °C and with a flow rate of 0.6 mL/min for 50 min. The chemical concentrations were monitored with a refractive index detector (RID) and quantified by comparing the peak areas to those of standard solutions.

### Oxygen consumption rate measurements

Oxygen consumption rates (OCRs) of YZy197 in media supplemented with different carbon sources were measured using a Seahorse XF96 Analyzer (Agilent Seahorse Bioscience, MA, USA) according to the manufacturer’s instructions. Yeast cells were grown to mid-log phase in SC-ura medium supplemented with different amounts (2%, 4%, 8%, 10%, or 15%) of xylose, glucose, or galactose. Cells were then washed and resuspended to OD_600_ of 1.0 in the fresh medium which was used for growth. 180 µL of resuspended cells was seeded in poly-l-lysine-coated XF96 plate via centrifugation (500*g* for 3 min) and then incubated for 30 min at 30 °C prior to measurement. The Seahorse XF96 sensor cartridge was sequentially hydrated at 30 °C with sterile water (overnight) and XF Calibrant (60 min) following the manufacturer’s instructions. The Seahorse XF96 Analyzer was set to maintain the temperature at 30 °C. Both the mixing time and measuring time were set to 3 min in each cycle.

## Supplementary information

**Additional file 1.** Additional Tables S1, S2 and Figures S1–S8.

## Data Availability

The authors declare that all data supporting the findings of this study are available within the paper (and its Additional files).

## Abbreviations

BCHAs: branched-chain higher alcohols
2-MbOH: 2-methyl-1-butanol
ALS: acetolactate synthase
KARI: ketol-acid reductoisomerase
DHAD: dehydroxyacid dehydratase
α-KIV: α-ketoisovalerate
α-KDCs: α-ketoacid decarboxylases
ADHs: alcohol dehydrogenases
XI: xylose isomerase
XR: xylose reductase
XHD: xylitol dehydrogenase
X5P: xylulose-5-phosphate
XK: xylulokinase
PPP: pentose phosphate pathway
