# Supplementary material for: Xylose utilization stimulates mitochondrial production of isobutanol and 2-methyl-1-butanol in Saccharomyces cerevisiae
Source: Biotechnol Biofuels. 2019 Sep 20;12:223. doi: 10.1186/s13068-019-1560-2 (PMC6753614; doi:10.1186/s13068-019-1560-2)
Supplement: Supplementary file 1 — Additional file 1. Additional Tables S1, S2 and Figures S1–S8. [file 13068_2019_1560_MOESM1_ESM.pdf]

Additional file to:

Xylose utilization stimulates mitochondrial production of isobutanol and 2-methyl-1-butanol in *Saccharomyces cerevisiae*

Yanfei Zhang [1], Stephan Lane [4, 5], Jhong-Min Chen [1], Sarah K. Hammer [1], Jake Luttinger [1], Lifeng Yang [6, 7], Yong-Su Jin [4, 5] and José L. Avalos [1, 2, 3, *]

[1] Department of Chemical and Biological Engineering; [2] Andlinger Center for Energy and the Environment; [3]Department of Molecular Biology, Princeton University, Princeton, NJ, USA; [4] Carl R. Woese Institute for Genomic Biology, University of Illinois at Urbana-Champaign, Urbana, IL, USA; [5]Department of Food Science and Human Nutrition, University of Illinois at Urbana-Champaign, Urbana, IL, USA; [6] Lewis Sigler Institute for Integrative Genomics, Princeton University, Princeton, NJ, USA; [7] Department of Chemistry, Princeton University, Princeton, NJ, USA

*Corresponding author: José L. Avalos

Department of Chemical and Biological Engineering,

Princeton University,

101 Hoyt Laboratory, William Street, Princeton, NJ 08544, USA

Phone office: +1 (609) 258-9881

Phone lab: +1 (609) 258-0542

Fax: +1 (609) 258-1247

Email: javalos@princeton.edu

Table S1. Final titers, overall yields, maximum daily yields, and maximum productivities of isobutanol, 2-methyl-1-butanol and total branched-chain higher alcohols in fermentations with different concentrations of xylose, glucose, or galactose.

	Isobutanol				2-methyl-1-butanol				Total branched-chain higher alcohols			
	Titer (g/L)	Overall Yield (mg/g Sugar)	Daily Yield (mg/g Sugar)	Productivity (mg/L/h)	Titer (g/L)	Overall Yield (mg/g Sugar)	Daily Yield (mg/g Sugar)	Productivity (mg/L/h)	Titer (g/L)	Overall Yield (mg/g Sugar)	Daily Yield (mg/g Sugar)	Productivity (mg/L/h)
4% Xylose	0.42 ± 0.02	10.5 ± 0.4	9.9 ± 1.6	16.4 ± 2.6	0.31 ± 0.03	7.7 ± 0.7	6.0 ± 0.2	9.9 ± 0.3	0.73 ± 0.05	18.2 ± 1.1	15.9 ± 1.8	26.3 ± 2.9
8% Xylose	1.20 ± 0.04	15.1 ± 0.4	27.6 ± 8.0	22.8 ± 4.0	0.72 ± 0.04	8.0 ± 0.1	9.9 ± 0.5	14.7 ± 1.5	1.92 ± 0.08	23.1 ± 0.5	37.5 ± 8.5	37.5 ± 5.5
10% Xylose	2.05 ± 0.21	20.7 ± 2.2	26.2 ± 5.5	28.1 ± 1.5	0.91 ± 0.02	9.2 ± 0.2	11.9 ± 1.6	15.8 ± 1.3	2.96 ± 0.23	29.9 ± 2.4	38.1 ± 7.1	43.9 ± 2.8
15% Xylose	2.72 ± 0.10	21.7 ± 0.4	25.8 ± 2.9	23.0 ± 1.5	0.86 ± 0.02	6.9 ± 0.2	12.2 ± 0.5	11.5 ± 0.5	3.58 ± 0.12	28.6 ± 0.6	38.0 ± 3.4	34.5 ± 2.0
10% Glucose	1.07 ± 0.01	10.8 ± 0.1	10.8 ± 0.1	26.7 ± 1.6	0.68 ± 0.05	6.8 ± 0.5	7.0 ± 0.2	17.7 ± 0.7	1.75 ± 0.06	17.6 ± 0.6	17.8 ± 0.3	44.4 ± 2.3
15% Glucose	1.55 ± 0.06	10.0 ± 0.2	12.6 ± 3.4	26.6 ± 1.2	0.75 ± 0.05	4.9 ± 0.4	6.9 ± 0.2	15.2 ± 3.5	2.30 ± 0.11	14.9 ± 0.6	19.5 ± 3.6	41.8 ± 4.7
10% Galactose	1.32 ± 0.12	13.4 ± 1.3	17.9 ± 7.7	12.2 ± 1.6	0.93 ± 0.16	9.4 ± 1.5	11.7 ± 1.8	9.1 ± 1.6	2.25 ± 0.28	22.8 ± 2.8	29.6 ± 9.5	21.3 ± 3.2
15% Galactose	1.81 ± 0.09	14.5 ± 0.8	18.4 ± 4.1	13.2 ± 0.2	0.94 ± 0.02	7.7 ± 0.1	11.8 ± 0.5	9.2 ± 0.3	2.75 ± 0.11	22.2 ± 0.9	30.2 ± 4.6	22.4 ± 0.5
10% Xylose-Fed*	2.96 ± 0.06	16.3 ± 0.5	23.3 ± 0.8	32.6 ± 1.7	0.84 ± 0.05	4.9 ± 0.4	12.4 ± 1.0	16.3 ± 1.4	3.80 ± 0.11	21.2 ± 0.9	35.7 ± 1.8	48.9 ± 3.1
15% Xylose-Fed*	3.10 ± 0.18	19.6 ± 0.8	38.8 ± 2.4	30.9 ± 0.7	0.79 ± 0.07	5.3 ± 0.4	18.4 ± 2.8	14.7 ± 1.6	3.89 ± 0.25	24.9 ± 1.2	57.2 ± 5.2	45.6 ± 2.3

* Fed-batch fermentation

Table S2. Oligonucleotides used in this study.

Oligo Name	Sequence	Note
Yfz_Oli67	TAATTACCGTTCGTATAATGTATGCTATACGAAGTTATTAGGTCTAGAGATCTGTTTAGC	Gibson primer to replace Lox66 in pUG6 F
Yfz_Oli68	GTATAGCATACATTATACGAACGGTAATTAAGGGTTGTCGACCTGCAGCGTACGAAGCTT	Gibson primer to replace Lox66 in pUG6 R
Yfz_Oli69	ATAACTTCGTATAATGTATGCTATACGAACGGTATAGGTGATATCAGATCCACTAGTGGC	Gibson primer to replace Lox71 in pUG6 F
Yfz_Oli70	TACCGTTCGTATAGCATACATTATACGAAGTTATATTAAGGGTTCTCGAGAGCTCGTTTT	Gibson primer to replace Lox71 in pUG6 R
Yfz_KO189	ACTACATGTTTTCGTTAGAATAAATCACCCTATAAACGCAAAATCAGCTAGAACCTTAGCATACTAAAACtacgctgcaggtcgacaacc	ScBAT1_KO_70bp_F
Yfz_KO190	AACAGATCCTCTGAGAGGAATTCTCGTTTTTTTTTTTGGGGGGGAGGGGATGTTTACCTTCATTATCActagtggatctgatatcacc	ScBAT1_KO_70bp_R
Yfz_KO245	TCTTGTTTTATAGAAGAAAAAACATCAAGAAACATCTTTAACATACACAAACACATACTATCAGAATACAtacgctgcaggtcgacaacc	ScALD6_KO_70bp_F
Yfz_KO246	GACGTAAGACCAAGTAAGTTTATATGAAAGTATTTTGTGTATATGACGGAAAGAAATGCAGGTTGGTACActagtggatctgatatcacc	ScALD6_KO_70bp_R
Yfz_KO233	ATCAAGCTCGAGCCAAATCACAAAAAAAGCCTTATAGCTTGCCCTGACAAAGAATATACAACTCGGGAAAtacgctgcaggtcgacaacc	ScPHO13_KO_70bp_F
Yfz_KO234	TAAAAACAACAAACCTGAATATTTTTCCTTTTCAAAAAGTAATTCTACCCCTAGATTTTGCATTGCTCCTctagtggatctgatatcacc	ScPHO13_KO_70bp_R

Underline indicates the annealing sequences primer 1 (5'-TACGCTGCAGGTCGACAACC-3'), and primer 2 (5'-CTAGTGGATCTGATATCACC-3').

Figure S1.

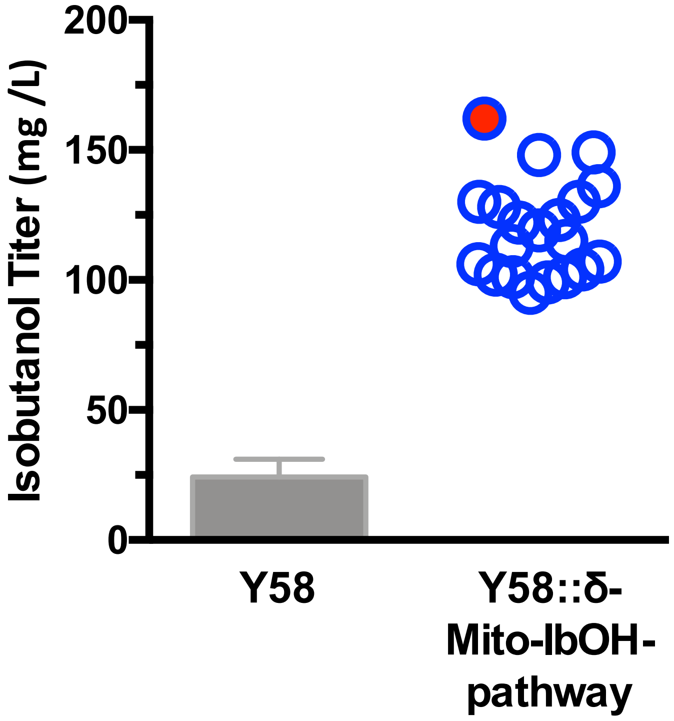

Figure S1. Screen of 20 colonies of Y58 transformed with the mitochondrial isobutanol production pathway integrated into δ-sites. High cell-density fermentations were carried out in 15% xylose in synthetic complete (SC) medium for 48 h. The strain with the highest isobutanol production (red) was isolated as YZy165 for future development and study. The data for Y58 represent the mean ± s.d. of biological triplicates.

Figure S2

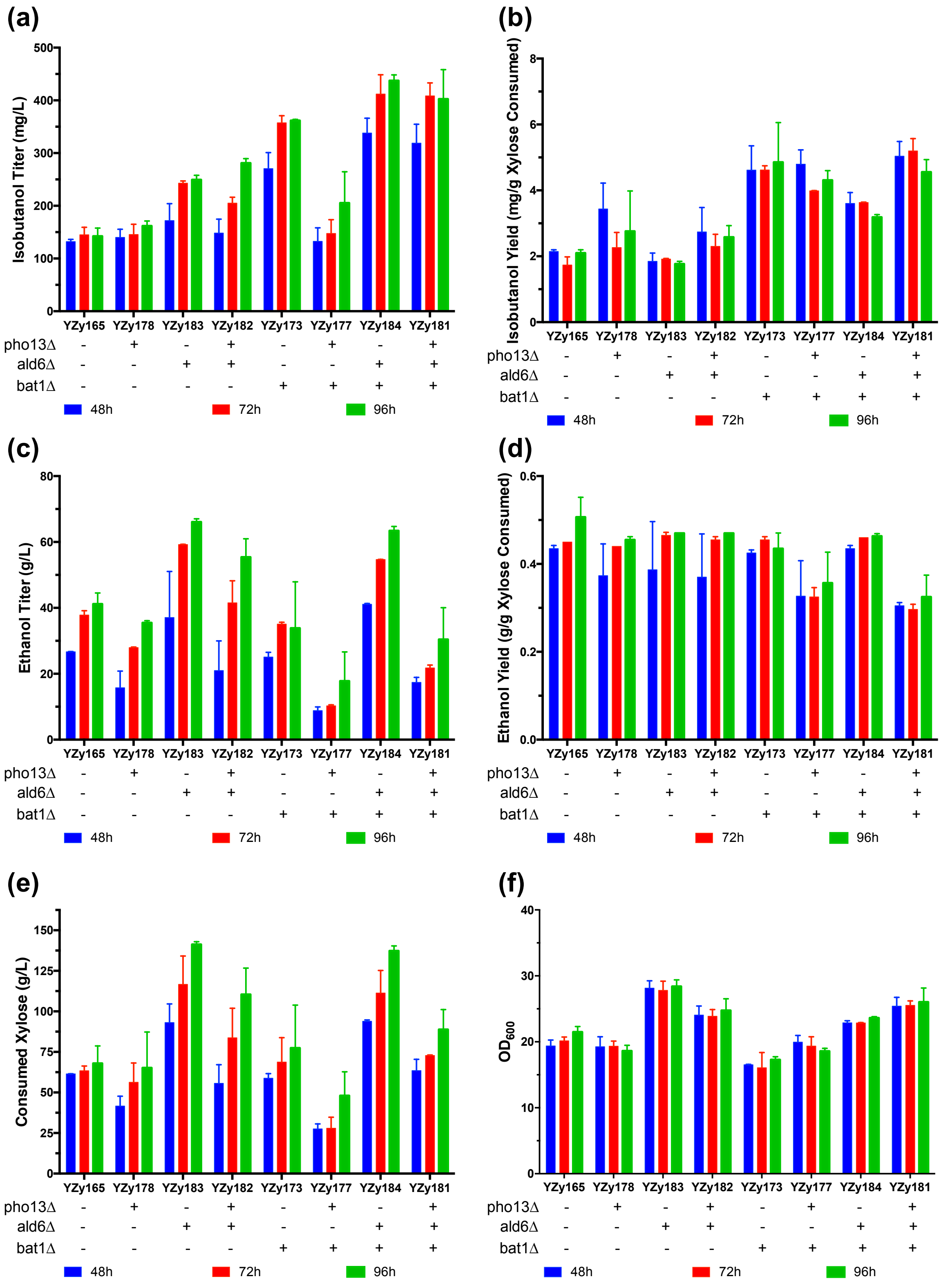
(a)
Isobutanol Titer (mg/L)
(b)
Isobutanol Yield (mg/g Xylose Consumed)
(c)
Ethanol Titer (g/L)
(d)
Ethanol Yield (g/g Xylose Consumed)
(e)
Consumed Xylose (g/L)
(f)
OD600
YZy165 YZy178 YZy183 YZy182 YZy173 YZy177 YZy184 YZy181
pho13Δ - + - + - + - +
ald6Δ - - + + - - + +
bat1Δ - - - - + + + +
48h
72h
96h

Figure S2. Time course fermentations of strains carrying gene deletions. Isobutanol titers (a) and yields (b); ethanol titers (c) and yields (d); xylose consumption (e) and OD_{600} (f) monitored at 48 h (blue), 72 h (red), and 96 h (green). All data represent the mean ± s.d. of biological triplicates.

Figure S3

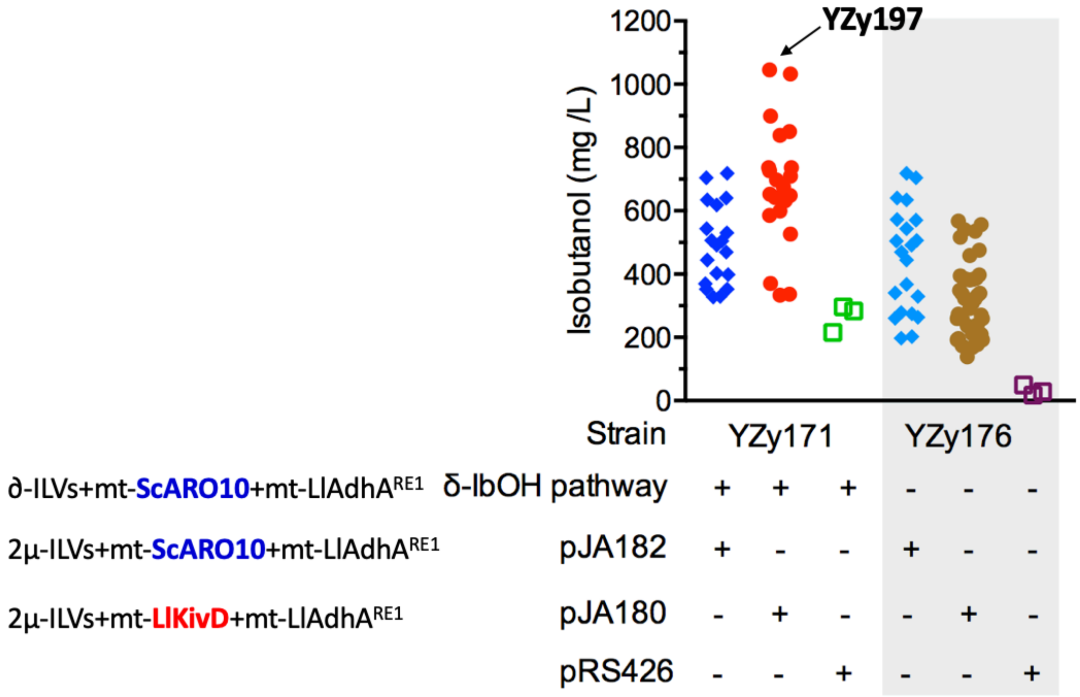

Figure S3. Isobutanol production from colonies of strains containing the isobutanol biosynthetic pathway integrated in δ-sites (YZy171) or not (YZy176) transformed with 2μ plasmids pJA182 (containing the *ILV* genes, CoxIV$_{MLS}$-*ARO10*, and CoxIV$_{MLS}$-LlAdhA^{RE1}), pJA180 (containing the *ILV* genes, CoxIV$_{MLS}$-LlKivd, and CoxIV$_{MLS}$-LlAdhA^{RE1}), or pRS426 (empty plasmid control). Each point represents the production from individual colonies of a transformation plate. The highest producer, YZy197, is marked with an arrow.

Figure S4

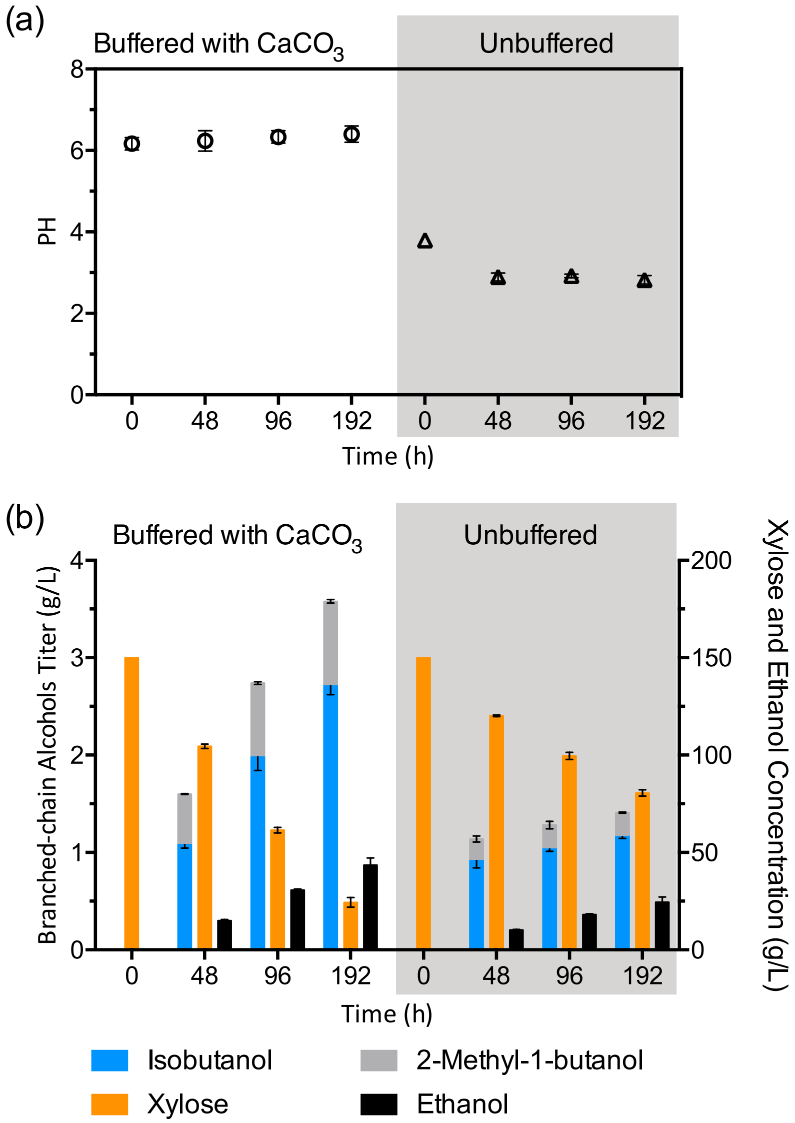

Figure S4. Fermentations in $CaCO_3$-buffered or unbuffered media for BCHA production from xylose. (a) Fermentation pH at different time points. (b) Concentrations of isobutanol, 2-methyl-1-butanol, xylose, and ethanol measured at time points 0 h, 48 h, 96 h and 192 h. Error bars represent the standard deviation of three independent fermentations.

Figure S5

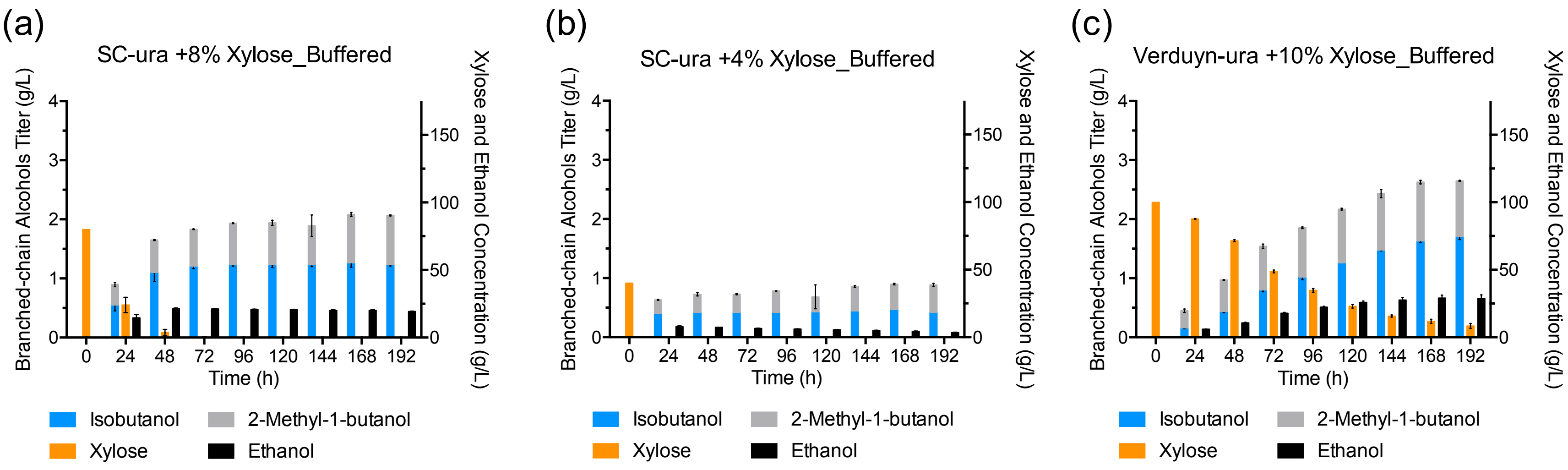

Figure S5. Time course of isobutanol, 2-MbOH, and ethanol production, as well as xylose consumption in YZy197 using synthetic complete medium with initial xylose concentration of 80 g/L (a), and 40 g/L (b); as well as in Verduyn's medium with initial xylose concentration of 100 g/L (c).

Figure S6.

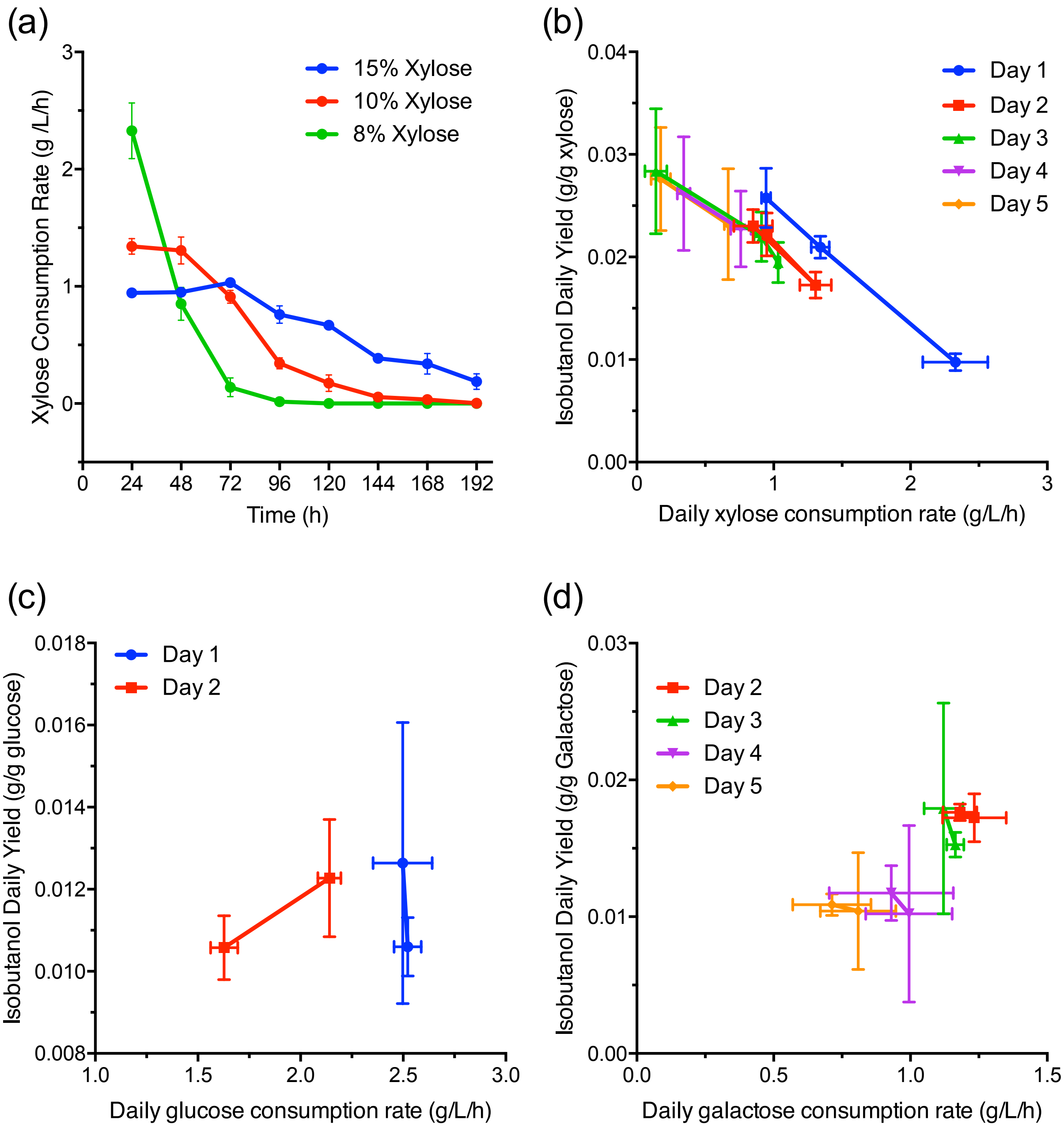

Figure S6. Effects of initial xylose concentrations and sugar consumption rates on isobutanol yields in YZy197 fermentations. (a) Effects of initial xylose concentration on daily xylose consumption rate in YZy197 fermentations. Y = $([Xyl]_{t=i-1}$ - $[Xyl]_{t=i})$/24h. [Xyl] = xylose concentration in g; and i = time point in daily (24 h) allotments. All data represent the mean ± s.d.

of biological triplicates. it is not possible to reliably measure xylose consumption rates in fermentations starting with 4% xylose, because the substrate is fully consumed within the first 24 hours, which is the shortest time point on our experiment. (b-d) Effects of sugar consumption rates on isobutanol yields in YZy197 fermentations. Daily Isobutanol yields at different consumption rates of xylose (b), glucose (c), and galactose (d), corresponding to different initial sugar concentrations of 15%, 10% and 8% (8% is only for xylose).

Figure S7

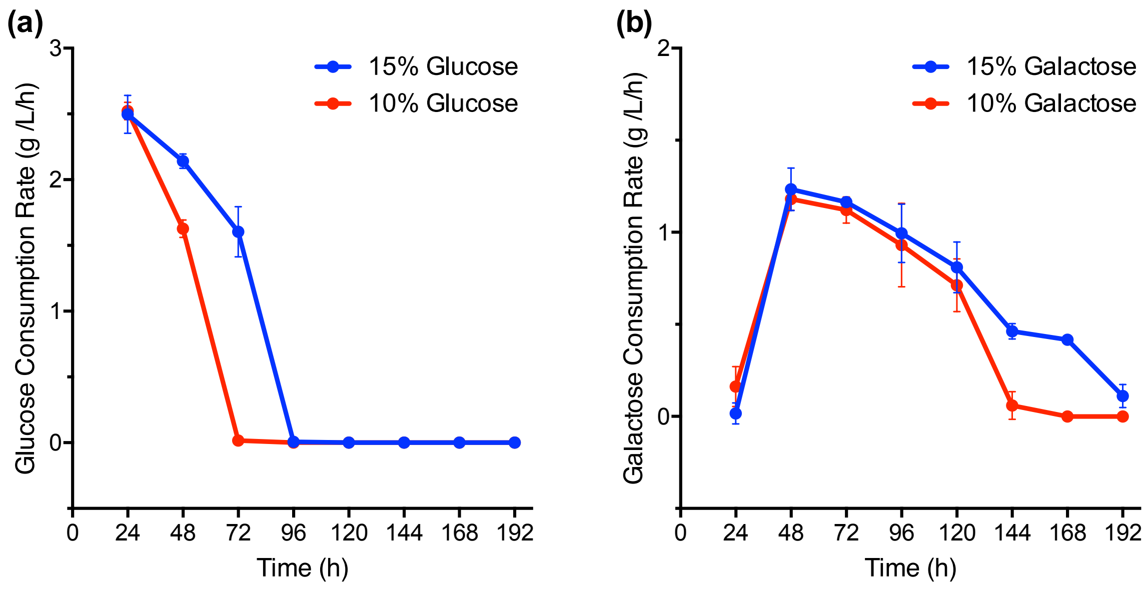

Figure S7. Daily hexose consumption rates at different initial sugar concentrations in YZy197 fermentations. Effects of initial glucose (a) or galactose (b) concentration on daily sugar consumption rate. $Y_{Glucose}$ = ([Glucose]$_{t=i-1}$ - [Glucose]$_{t=i}$)/24h; $Y_{Galactose}$ = ([Galactose]$_{t=i-1}$ - [Galactose]$_{t=i}$)/24h. [Glucose] = Glucose concentration in g; [Galactose] = Galactose concentration in g; and i = time point in daily (24h) allotments. All data represent the mean ± s.d. of biological triplicates.

Figure S8

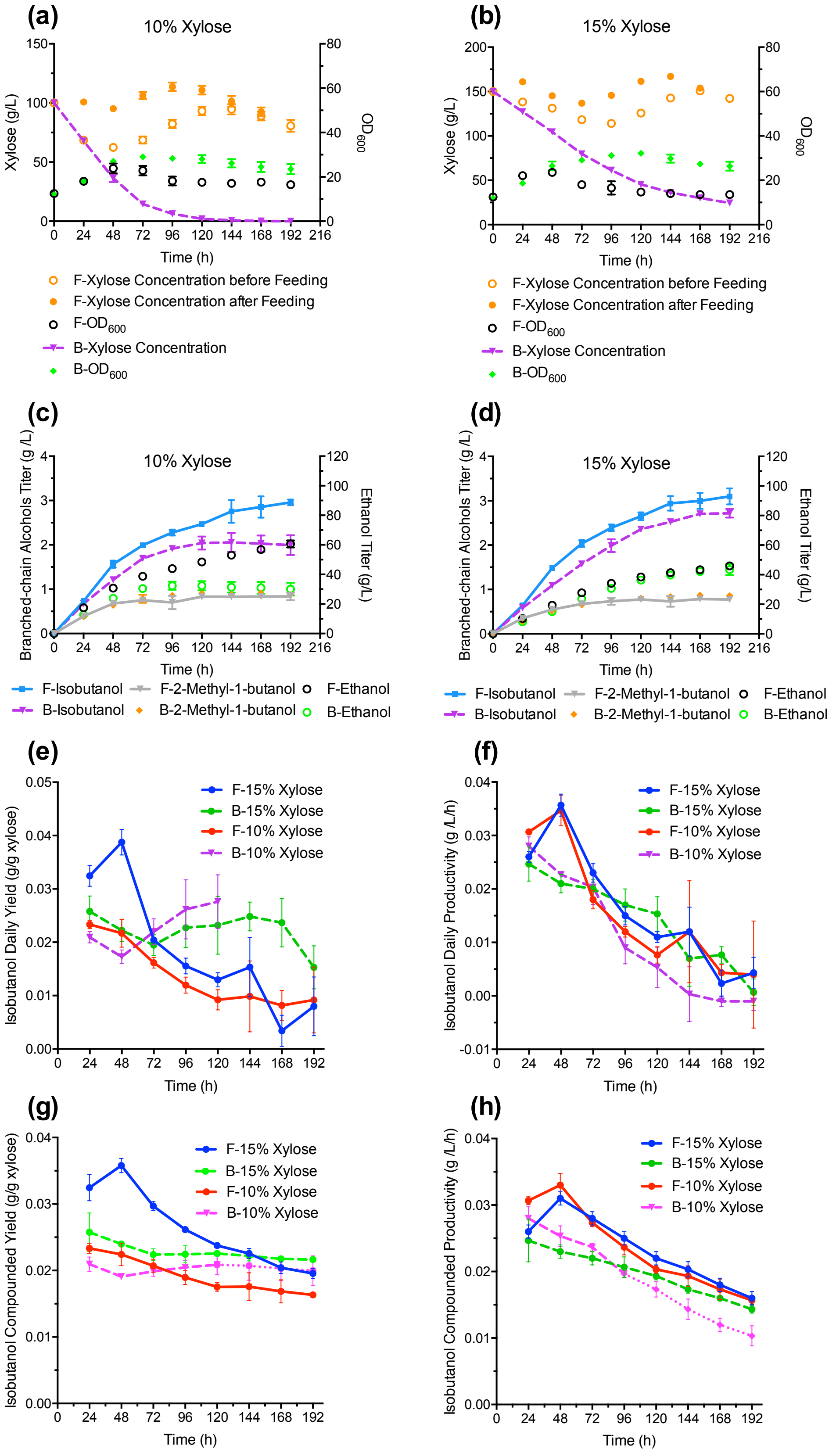
(a)
10% Xylose
Xylose (g/L)
OD600
Time (h)
F-Xylose Concentration before Feeding
F-Xylose Concentration after Feeding
F-OD600
B-Xylose Concentration
B-OD600
(b)
15% Xylose
Xylose (g/L)
OD600
Time (h)
F-Xylose Concentration before Feeding
F-Xylose Concentration after Feeding
F-OD600
B-Xylose Concentration
B-OD600
(c)
10% Xylose
Branched-chain Alcohols Titer (g /L)
Ethanol Titer (g/L)
Time (h)
F-Isobutanol
F-2-Methyl-1-butanol
F-Ethanol
B-Isobutanol
B-2-Methyl-1-butanol
B-Ethanol
(d)
15% Xylose
Branched-chain Alcohols Titer (g /L)
Ethanol Titer (g/L)
Time (h)
F-Isobutanol
F-2-Methyl-1-butanol
F-Ethanol
B-Isobutanol
B-2-Methyl-1-butanol
B-Ethanol
(e)
Isobutanol Daily Yield (g/g xylose)
Time (h)
F-15% Xylose
B-15% Xylose
F-10% Xylose
B-10% Xylose
(f)
Isobutanol Daily Productivity (g /L/h)
Time (h)
F-15% Xylose
B-15% Xylose
F-10% Xylose
B-10% Xylose
(g)
Isobutanol Compounded Yield (g/g xylose)
Time (h)
F-15% Xylose
B-15% Xylose
F-10% Xylose
B-10% Xylose
(h)
Isobutanol Compounded Productivity (g /L/h)
Time (h)
F-15% Xylose
B-15% Xylose
F-10% Xylose
B-10% Xylose

Figure S8. Comparison of fed-batch (F) and batch (B) fermentations. (a, b) Xylose concentration and cell optical density in OD_{600} during fermentation. In fed-batch fermentations (F) xylose was added every 24 hours to keep the concentration at approximately 10% (103 ± 8 g/L, a) and 15% (154 ± 11 g/L, b). Batch fermentations (B) were initiated at 100 g/L or 150 g/L of xylose. (c, d) Time course of isobutanol, 2-MbOH, and ethanol production in YZy197 using synthetic complete medium with maintained (F) or initial (B) xylose concentration of 100g/L (a) or 150g/L (b). (e) Isobutanol daily yields at different xylose concentrations: $Y_d = ([IbOH]_{t=i} - [IbOH]_{t=i-1})/([Xyl]_{t=i-1} - [Xyl]_{t=i})$. (f) Isobutanol daily productivities: $P_d = ([IbOH]_{t=i} - [IbOH]_{t=i-1})/24h$. (g) Isobutanol compounded yields at different xylose concentrations: $Y_c = [IbOH]_t/([Xyl]_{t=0} - [Xyl]_t)$. (h) Isobutanol compounded productivities: $P_c = [IbOH]_t/t$. [IbOH]. Where [IbOH] = isobutanol concentration in mg; [Xyl] = xylose concentration in g; i = time point intervals of 24 hours (daily), and t = time in hours. All data represent the mean ± s.d. of biological triplicates.
